# Ion channels as a therapeutic target for renal fibrosis

**DOI:** 10.3389/fphys.2022.1019028

**Published:** 2022-10-05

**Authors:** Peng Yan, Ben Ke, Xiangdong Fang

**Affiliations:** Department of Nephrology, The Second Affiliated Hospital of Nanchang University, Nanchang, China

**Keywords:** ion channels, renal fibrosis, kidney, Cl^−^ channels, Ca^2+^ signaling, K^+^ channels, Na^+^ transport

## Abstract

Renal ion channel transport and electrolyte disturbances play an important role in the process of functional impairment and fibrosis in the kidney. It is well known that there are limited effective drugs for the treatment of renal fibrosis, and since a large number of ion channels are involved in the renal fibrosis process, understanding the mechanisms of ion channel transport and the complex network of signaling cascades between them is essential to identify potential therapeutic approaches to slow down renal fibrosis. This review summarizes the current work of ion channels in renal fibrosis. We pay close attention to the effect of cystic fibrosis transmembrane conductance regulator (CFTR), transmembrane Member 16A (TMEM16A) and other Cl^−^ channel mediated signaling pathways and ion concentrations on fibrosis, as well as the various complex mechanisms for the action of Ca^2+^ handling channels including Ca^2+^-release-activated Ca^2+^ channel (CRAC), purinergic receptor, and transient receptor potential (TRP) channels. Furthermore, we also focus on the contribution of Na^+^ transport such as epithelial sodium channel (ENaC), Na^+^, K^+^-ATPase, Na^+^-H^+^ exchangers, and K^+^ channels like Ca^2+^-activated K^+^ channels, voltage-dependent K^+^ channel, ATP-sensitive K^+^ channels on renal fibrosis. Proposed potential therapeutic approaches through further dissection of these mechanisms may provide new therapeutic opportunities to reduce the burden of chronic kidney disease.

## 1 Introduction

Renal fibrosis is a process described by excessive proliferation of fibroblasts and deposition of extracellular matrix (ECM), which collectively lead to a broad maladaptive repair of affected renal tissue (Duffield, 2014). Under pathological conditions, the fibrotic process is triggered and coordinated by cross-talk between multiple cell types (Gewin et al., 2017). In particular, myofibroblasts, which are a key population for interstitial collagen matrix deposition, typically have the properties of contraction, proliferation, enhanced secretion, and expression of α smooth muscle actin (αSMA), a cytoskeletal protein of highly contractile microfilaments. In addition to primarily resident mesenchymal cells (fibroblasts and pericytes), other sources of myofibroblasts include perivascular fibroblasts, circulating fibroblasts, and epithelial-mesenchymal transition (EMT) transdifferentiated renal tubular epithelial cells (Mack and Yanagita, 2015). Interstitial inflammatory cell infiltration is also one of the characteristic histological features of renal fibrosis, which is observed in almost all types of renal disease and fibrosis (Huen and Cantley, 2017). Activated lymphocytes and macrophages as well as damaged epithelial cells secrete a variety of pro-inflammatory and pro-fibrotic factors, among which transforming growth factor-β (TGF-β) is recognized to be particularly prominent in renal fibrosis [for more factors references (Meng et al., 2016; Gewin et al., 2017)]. These cytokines promote the initiation of the fibrotic response by favoring fibroblast activation, inflammatory cell recruitment and endothelial cell loss, and achieve crosstalk between these cells. New evidence suggests that endothelial cell damage may be involved in endothelial-to-mesenchymal transition as well as in complex secretory synthesis (Lipphardt et al., 2017; Yang et al., 2019a). In addition, the kidney is a highly vascularized organ, and dysfunction of the interstitial capillary network mediating hypoxia and oxidative stress production is one of the important mechanisms driving renal fibrosis. Furthermore, when repeated epithelial injury occurs, apoptosis of renal tubular cells can develop, often resulting in tubular atrophy at a later stage. Interstitial fibrosis and tubular atrophy are common endpoints in almost all forms of kidney injury. Although a plethora of literature with in-depth and extensive mechanistic research, unfortunately there is no effective treatment for renal fibrosis, and the only proven method to slow the decline of renal function remains blockade of the renin-angiotensin system with angiotensin converting enzyme inhibitors (ACEI), angiotensin receptor blockers, or renin inhibitors (Francois and Chatziantoniou, 2018). Late development to end-stage renal disease (ESRD) still requires replacement therapy.

Ion channels are a class of pore-forming proteins found in all living cells that provide energetically favorable passage for ions to diffuse rapidly and passively according to their electrochemical potential (Roux, 2017). By mediating the influx or efflux of essential ions transported across the cell membrane, they can modulate the cytoplasmic or extracellular ion concentration, membrane potential and cell volume, which are fundamental to the survival and functional state of all cells. Regulation of changes in ion fluxes and channel activity in response to changing environmental requirements and stimuli is required for processes including proliferation, apoptosis, invasion, secretion and migration, and other cellular behavioral processes. Ions play an important role in different signaling pathways, since many extracellular molecules target ions for related functions, and in the last decade or so, the field of ion channels has developed rapidly. Indeed, the term “channelopathy” has been extended to describe a growing range of diseases associated with ion channel dysfunction. Dysregulated ion channels are involved in pathological conditions including hypertension, hyperglycemia, obesity, electrolyte disturbances, and cellular mechanical changes, all of which accelerate renal fibrosis phenotypes. Although the pathological factors and mechanisms of renal fibrosis remain incompletely understood, increasing evidence suggests that gene expression and signaling pathway activation associated with renal fibrosis are closely linked to the regulation of voltage- and non-voltage-gated ion channels. In this review, we summarize most recent data regarding the involvement of four major classes of Cl^−^, Ca^2+^, Na^+^, and K^+^ ion channels in the regulation of renal fibrosis. Continued efforts to explore their interactions and mechanisms, and to assess as candidate pharmacological targets of delay progression of renal failure will help develop more effective treatments.

## 2 Chloride channels

Cl^−^ channels are a group of functionally and structurally diverse anion-selective channels that have recently gained a considerable amount of interest. In the past, it was always thought that Cl^−^ was in electrochemical equilibrium on the membrane due to less research and the high resting Cl^−^ permeability, but it is now clear that in most cells, Cl^−^ is actively transported and out of electrochemical equilibrium (Duran et al., 2010), for example, some Cl^−^ channels or transporters like Cl^−^-HCO_3_^−^ exchangers, Na^+^-Cl^−^ cotransporters pump Cl^−^ into the cell, thus able to work and signal (Schmick and Bastiaens, 2014). Cl^−^ channels exhibit regulation of various physiological functions, including fluid secretion, cell volume, intracellular pH, and are involved in processes such as proliferation, trans-epithelial transport, cell cycle and electrical excitability (Stauber et al., 2012). Different Cl^−^ channels have been described in several categories based on structural and biological properties and gating characteristics: cystic fibrosis transmembrane conductance regulator (CFTR); Ca^2+^-activated Cl^−^ channels; voltage-activated Cl^−^ channels; volume-regulated anion channels (VRAC), ligand-gated channels, and other chloride channels (Suzuki et al., 2006). Here we focus on the most recent data regarding the involvement of these Cl^−^ channels in the behavior of renal fibrosis.

### 2.1 Cystic fibrosis transmembrane conductance regulator(CFTR)

CFTR, a member of the ATP-binding cassette transporter protein superfamily, is widely expressed in apical epithelial membranes including the kidney, lung, liver, and reproductive tract (Csanady et al., 2019). CFTR is a cAMP-dependent anion channel consisting of a dimer with six transmembrane structures per subunit that regulate fluid transport and electrolyte balance. Dysfunction of CFTR leads to abnormal anion secretion, causing a series of epithelial dysfunctions and the development of chronic inflammatory. Cystic fibrosis (CF) is the result of a CFTR mutation, which is well described in terms of lung symptoms (Gibson et al., 2003). Some studies have shown that CFTR has a tumor suppressive effect in various types of cancer (Maisonneuve et al., 2013; Liu et al., 2020). In addition, CFTR is abundantly expressed on the apical surface of renal tubules, and CF patients had more prominent proteinuria, which may be caused by tubular dysfunction and interstitial injury (Jouret and Devuyst, 2009), thus suggesting that CFTR is closely associated with renal fibrotic disease.

Functional CFTR deficiency result in epithelial cells transforming into a more proliferative, less differentiated state that is more sensitive to EMT stimuli such as TGF-β1. Mesenchymal markers such as N- cadherin, vimentin, collagen I, and fibronectin are significantly upregulated in the native human CF airways compared to non CF airways (Quaresma et al., 2020), and increase CFTR activity using the potent CFTR modulator (HECM) drug restores the mutated CFTR-induced epithelial phenotype and confers direct protection against EMT (Narayanan et al., 2020). In the kidney, the mouse δF508 CFTR mutation exacerbates the fibrotic phenotype induced by unilateral ureteral obstruction (UUO), which is a well-established animal model of renal fibrosis, and *in vitro*, inhibition of CFTR activity using the inhibitors inh-172 or GlyH101 is sufficient to trigger the EMT process in renal cells (Zhang et al., 2017). In addition, it has been recognized that CFTR deficiency may lead to disruption of the cytoskeleton and reduced formation of cellular tight junctions in renal tubular epithelial cells by reducing direct Zonula Occludens-1 (ZO-1) interactions (Ruan et al., 2014; Castellani et al., 2017), which is one of the characteristics of EMT. Yes-associated protein 1 (YAP1) is an important mammalian transcriptional effector regulating the Hippo pathway and has been shown to play a key role in organ development, fibrosis, and wound healing (Dey et al., 2020). YAP1 was found to be aberrantly active in the presence of mutant CFTR, and is an important mediator of CFTR-related fibrosis/EMT processes (Quaresma et al., 2022). However, further elaboration is needed regarding its more precise mechanism.

**FIGURE 1:**
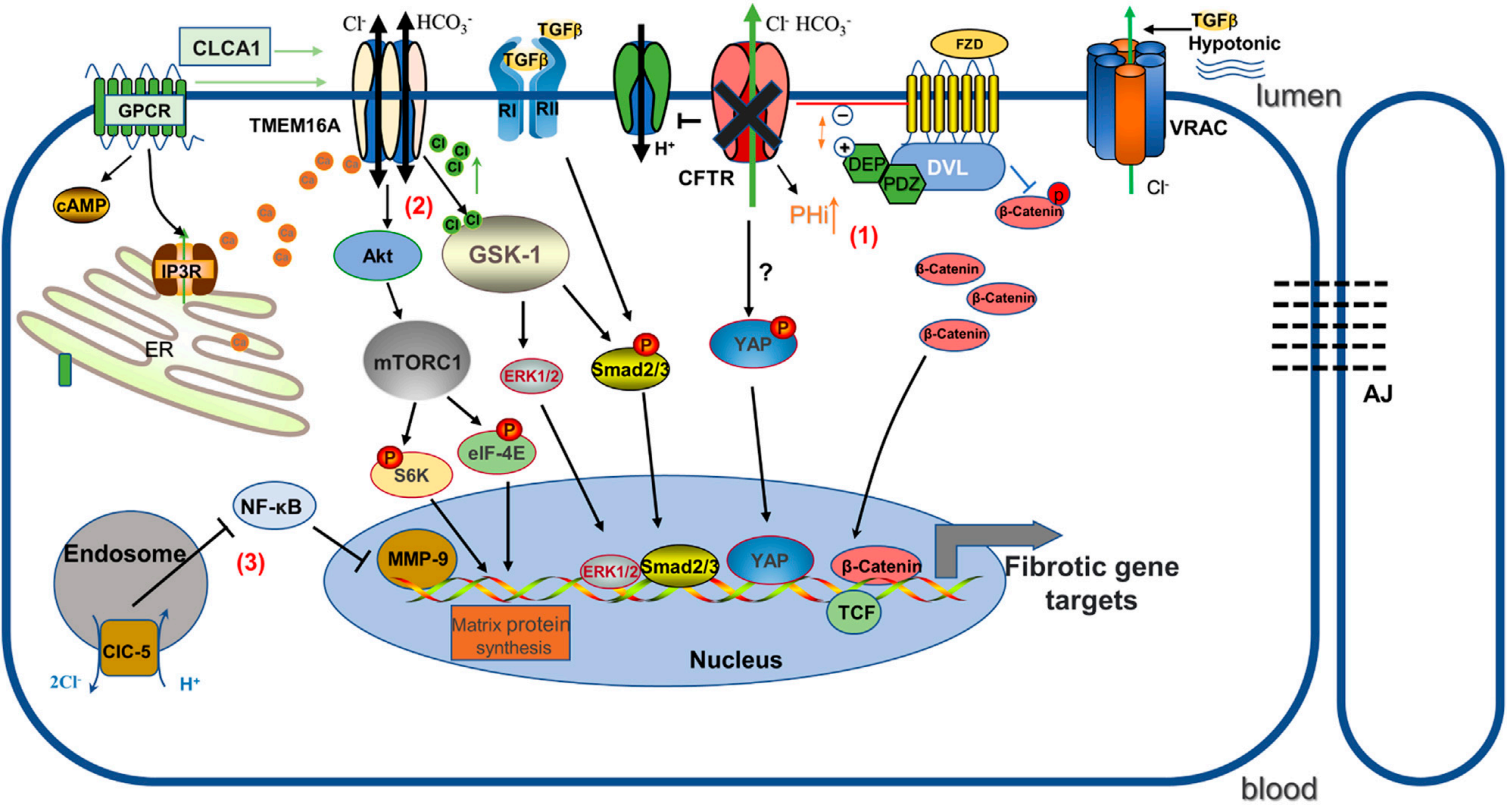
Schematic diagram illustrating the mechanisms by which several chloride channels affect gene expression in renal fibrosis. (1) CFTR deficiency causes increased intracellular pH. It promotes the binding of positively charged DEP and negatively charged phospholipids at the plasma membrane, and as a result, increased association of FZD and Dvl inhibits the phosphorylated degradation of β-catenin and promotes Wnt/β-catenin signaling. In addition, YAP may play a role in promoting renal fibrosis as one of the mechanisms. (2) TMEM16A stimulated by chloride channel accessory 1 (CLCA1) activates mTORC1-mediated matrix protein synthesis, and GSK-1 induces pro-fibrotic expression in response to TMEM16A-induced activation by increased Cl-concentration. (3)ClC-5 overexpression inhibits NF-κB/MMP-9. FZD: Frizzled; DVL: Dishevelled; AJ: Adherens junctions; ClC-5: voltage-gated chloride channel-5.

CFTR dysfunction activates canonical Wnt/β-catenin signaling to mediate tubular epithelial-mesenchymal fibroblast transition (Zhang et al., 2017). Wnt/β-catenin is an evolutionary conserved signaling pathway that regulates cell fate, homeostasis and regeneration, and is activated following kidney injury in various animal models and human kidney diseases. β-catenin overexpression induces fibrotic features, including epithelial cell dedifferentiation and EMT (Clevers and Nusse, 2012). Interestingly, CFTR expression was downregulated in the UUO mouse model and human fibrotic kidney, and the UUO-induced deltaF508 fibrosis mouse model has significantly higher β-catenin protein activity and renal fibrosis aggregation, which can be rescued by overexpression of CFTR, implying that CFTR regulation appears to be a potential therapeutic target for anti-fibrosis *via* Wnt/β-catenin signaling (Zhang et al., 2017). This result was further demonstrated by Liu and his colleagues in a rat model of diabetic nephropathy (Liu et al., 2021). Mechanistically, CFTR can interact with Dishevelled2 (Dvl2), a vital component of Wnt signaling, to inhibit β-catenin activation *via* the PDZ structural domain, as Dvl2 has PDZ domain and CFTR has a PDZ binding domain (Simons et al., 2009; Zhang et al., 2017). However, the nature of how this interaction inhibits β-catenin activity has not been fully elucidated. Moreover, it may also be related to the pH and charge changes of the Wnt pathway. Loss of CFTR function leads to elevated intracellular PH due to impaired Cl^−^ and HCO_3_^−^ secretion and dysregulation of the H^+^ pump (membrane potential changes induced by Cl^−^ flux contribute to the acidification of H^+^-ATPase) (Massey et al., 2021). It has been shown that intracellular alkalinization enhances the interaction of DvL with the Wnt receptor Frizzled proteins (FZD) by addressing DvL to negatively charged phospholipids on the plasma membrane through its positively charged DEP structural domain, thereby inhibiting β-catenin phosphate degradation and promoting Wnt signaling (Simons et al., 2009) (see Figure 1).

Notably, CFTR positively regulates β-catenin protein activity in the hematopoietic system and embryonic stem cells (Liu et al., 2017; Sun et al., 2018), implying that CFTR seems to respond differently to β-catenin signaling in cellular context, possibly by acting on different components of the Wnt/β-catenin pathway.

With more clinical focus regarding CFTR in autosomal dominant polycystic kidney disease (ADPKD), the importance of CFTR in fibrosis is increasingly recognized. Increased cAMP levels stimulate apical CFTR choreography, which contributes to epithelial cell proliferation and Cl^−^-dependent fluid secretion, while ECM gradually accumulates in the kidney with cystogenesis and the production of pro-fibrotic factors (Ramasubbu et al., 1998; Norman, 2011). Moreover, the cyst wall contains an extensive and abnormal capillary network and the absence of peritubular capillaries is a feature of tubulointerstitial fibrosis in chronic kidney disease (CKD). While deeply investigating the role of CFTR in kidney disease, independently of ADPKD, applying this target to chronic kidney disease or fibrotic kidney disease would be quite an attractive therapeutic treatment in terms of prospect.

### 2.2 Transmembrane member 16A

Transmembrane member (TMEM) 16A (also known as Anoctamin-1,ANO1), a Ca^2+^-activated Cl^−^ channel, belongs to the 10-member of the TMEM16 family and is known to transport chlorine and bicarbonate (Pedemonte and Galietta, 2014). Structurally TMEM16A is a revealed homodimer membrane protein with each subunit containing a ten-transmembrane helix structure (TM1-10), in which an ion-selective pore and two Ca^2+^-sensitive binding sites were identified. When Ca^2+^ directly binds to a site TM6-8 located near the cytoplasmic end of the membrane pore, the TM6 conformation is triggered to change, causing the pore to dilate and thus leaving the channel in an open state (Dang et al., 2017; Paulino et al., 2017). TMEM16A is widely expressed in various cells and mediates a variety of fundamental physiological functions, such as epithelial regulation-secretion, smooth muscle contraction, cell volume regulation, cell proliferation and sensory transduction (Yang et al., 2008; Hartzell et al., 2009; Oh and Jung, 2016). TMEM16A activity may be involved in the regulation of epithelial CFTR-dependent Cl^−^ transport. Recently, information about the relationship between TMEM16A and renal fibrosis was reported.

TMEM16A contributes to renal fibrosis through increased intracellular Cl^−^ concentration and TGF-β1-dependent pathways (Li et al., 2022a). Previous studies have indicated that TMEM16A expression is strongly expressed in the kidneys of patients with IgA nephropathy, renal cyst models and mice with high-fat diet/streptozotocin-induced diabetic nephropathy (Buchholz et al., 2014; Lian et al., 2017; Li et al., 2022a), suggesting an important role for TMEM16A in kidney disease. Similarly, Li et al. found that TMEM16A expression was markedly increased in fibrotic kidneys from UUO and high-fat diet mouse models, and that inhibition of TMEM16A activity *in vivo* with specific inhibitors or knockdown by shRNA effectively attenuated UUO-induced renal fibrosis and macrophage infiltration (Li et al., 2022a). Also, in cultured human proximal renal tubular epithelial (HK2) cells, inhibition of TMEM16A effectively reduced TGF-β1-induced EMT and restored E-cadherin abundance. Mechanistically, high levels of TMEM16A respond to TGF-β1-induced increases in [Cl]i, and mediate the pro-fibrotic effects of TGF-β1 in a Cl^−^ sensitive serum- and glucocorticoid-inducible protein kinase 1 (SGK1) dependent manner through the intracellular effectors Smad2/3 and extracellular signal-regulated kinases 1 and 2 (ERK1/2) pathways (Li et al., 2022a) (Figure 1). However, we recognize that TMEM16A is an outward rectifier current that promotes Cl^−^ secretion. The mechanism regarding TMEM16A mediating the elevation of [Cl]i in renal fibrosis has not yet been fully elucidated, and may be due to 1) differences in Cl^−^ concentration on renal tubular epithelial cells that predispose to inward flow, 2) indirect regulation of other Cl^−^ reabsorption pathways, 3) pathological conditions may shift Cl^−^ outflow to influx when the membrane potential reaches the Cl^−^ reverse potential (Deba and Bessac, 2015). Notably, nephron-specific TMEM16A knockout mice cause reduced glomeruli numbers and subsequently albuminuria and tubular injury (Faria et al., 2014; Schenk et al., 2018), which may be related to TMEM16A regulation of albumin uptake and endosomal acidification functions (Faria et al., 2014). Therefore, TMEM16A contributes to the regulation of renal function, but also plays an important role in response to environmental stimuli such as obstruction and high fat in renal injury.

Recently, an unexpected association between TMEM16A and senescence-associated secretory phenotype (SASP)-associated renal fibrosis was found. Aging kidney injury is an important driver of interstitial fibrosis, in part because senescent cells secrete important pro-inflammatory and pro-fibrotic mediators in the senescence-associated secretory phenotype, which directly affects the surrounding microenvironment, thereby triggering the persistent fibrotic process (Docherty et al., 2019; Docherty et al., 2020). Cellular senescence can therefore be considered as a pathological feature of renal fibrosis. It was found that chloride channel accessory 1 (CLCA1) expression was increased in aged mice and its overexpression enhanced TMEM16A activity in a paracrine manner (Sala-Rabanal et al., 2015), subsequently activating mTORC1 in the process. Activation of mTORC1 signaling is not only involved in renal fibroblast activation and collagen synthesis, but also increases the expression of TGF-β1, which mediates the development of fibrosis, and finally mTORC1 regulates oxidative stress injury and stem cell failure to accelerate cell and tissue aging (Zoncu et al., 2011; Jiang et al., 2013; Fantus et al., 2016). Inhibition of TMEM16A reduced mTORC1 activation and matrix protein synthesis in CLCA1 overexpressing cells, while also inhibiting SASP (Lee et al., 2021). Based on the above findings, TMEM16A is considered a potential target to prevent senescence-associated renal injury. However, the mechanism of how the process of cell surface anion secretion should trigger SASP remains unclear.

In diabetic nephropathy, TMEM16A activation also promoted podocyte apoptosis in diabetic mice by activating the P38/c-jun N-terminal kinase (JNK) signaling pathway (Lian et al., 2017). In conclusion, the important role of TMEM16A in promoting kidney injury through the regulation of multiple signaling pathways suggests that is TMEM16A may be a potential new molecular target for preventing the progression of renal fibrosis and chronic kidney disease.

### 2.3 Voltage-gated Cl^−^ channels

The voltage-gated chloride channel (ClC) family is a class of dimers consisting of nine isomers in mammals further divided into three groups: 1) ClC-1, ClC-2, ClC -Ka, and ClC -Kb; 2) ClC-3 to ClC-5; and 3) ClC-6 and ClC-7 (Jentsch and Pusch, 2018). It is found that ClC-3 to ClC-7 are mainly present in the endolysosomal membrane where they actually function as 2 Cl^−^/H^+^ exchangers, whereas the other types are plasma membrane Cl^−^ channels (Picollo and Pusch, 2005; Scheel et al., 2005; Jentsch and Pusch, 2018). By exchanging chlorine for hydrogen, ClCs provide a Cl^−^ shunt conductance in the lysosomes to neutralize the positive charge and promotes acidification (Gunther et al., 2003; Hara-Chikuma et al., 2005). Recently, the role of ClC in renal fibrosis has been gradually recognized.

As a members of the ClC superfamily, ClC-5 is an intracellular Cl^−^ channel encoded by the chloride voltage-gated channel 5 (CLCN5) gene, which is involved in cell proliferation, apoptosis, cellular electrical activity and volume homeostasis in addition to controlling acidification (Devuyst and Luciani, 2015). Like all other eukaryotic ClCs, ClC-5 is homodimeric with 18 transmembrane domains per subunit containing an independent ion-permeable pore where it is labeled by three anion-binding sites (Dutzler, 2004). ClC-5 is highly expressed in different renal tubular segments as well as in podocytes, and is upregulated in glomeruli of proteinuric nephropathy patients, suggesting that ClC-5 may play a role in the formation of proteinuric nephropathy (Ceol et al., 2012; Solanki et al., 2018). Previous studies indicated that aging ClC-5 knockout mice significantly increased renal tubular atrophy, interstitial fibrosis, renal calcinosis and had elevated TGF-β1 compared to wild-type mice, and that high citrate food feeding protected renal function and delayed the progression of renal disease (Cebotaru et al., 2005). In contrast, Yang’s group showed that mice with ClC-5 upregulation using a specialized adeno-associated virus vector largely protected against the development of renal fibrosis and inflammatory lesions after unilateral ureteral obstruction (UUO), and restored E-cadherin synthesis and reduced vimentin, α-SMA, and collagen fibril expression in the renal cortex (Yang et al., 2019b). Moreover, in renal tubular epithelial cells, ClC-5 overexpression prevented the epithelial-to-mesenchymal transition induced by TGF-β1 and matrix metalloproteinase-9 (MMP-9) expression (Yang et al., 2019b). Matrix metalloproteinases (MMP) are a large class of proteins that require metal ions as active forms of cofactors and have different roles in different pathological conditions (Zitka et al., 2010). MMP-9 has been shown to be critically involved in the pro-fibrotic microenvironment in the obstructed kidney by promoting growth factor release and communication between the epithelial and interstitial compartments (Tan et al., 2010; Wang et al., 2019a). ClC-5 expression inhibits immune cell infiltration and inflammatory cytokine release and ameliorates renal fibrosis by inhibiting NF-κB-mediated activation of MMP-9 pathway signaling (Yang et al., 2019b) (see Figure 1).

Interestingly, MMP9 appears to have a strong positive correlation with ClC-3, which regulates the extracellular environment and promotes the migration and invasion of cancer cells through multiple pathways of upregulation of MMP9 expression (Guan et al., 2016; Wang et al., 2017; Guan et al., 2019). ClC-3 and ClC-5 are very similar in basic biophysical properties, both of them are expressed intracellularly in almost all cell types and have been shown by several studies to be actually Cl^−^/H^+^ reverse transporter proteins, but ClC-3 is more widely distributed compared to ClC-5 (Steinmeyer et al., 1995). In human keratinocytes and human fetal lung fibroblasts, ClC-3 overexpression showed significantly more α-smooth muscle actin (α-SMA) expression as well as increased myofibroblast activation (Yin et al., 2008), suggesting a role for ClC-3 in fibroblast transformation. It is well known that renal oxidative stress production is the key to the development of renal fibrosis. Although ClC-3 is mainly expressed in endosomes and lysosomes, extracellular production of superoxide flux can mediate intracellular signaling through plasma membrane ClC-3 channels, further activating the production of mitochondrial reactive oxygen species (ROS) (Hawkins et al., 2007; Jha et al., 2016). However, the exact role and mechanism of ClC-3-mediated renal oxidative stress and fibrosis remains unclear. Because ClC-3 or ClC-5 are expressed primarily on organelle membranes, it is difficult to record their currents, and thus many of their proposed roles in a variety of biological processes may need to be reevaluated, although it is an interesting and relatively unexplored target in renal fibrosis.

It is worth mentioning that other ClC members, such as ClC-2 channels promote the migration transition of myofibroblasts and ECM synthesis (collagen I and fibronectin) *via* PI3K/Akt signaling (Sun et al., 2016), and that deletion of ClC2 alters the integrity of adherens junctions, leading to the release of membrane-bound β-catenin and activation of Wnt target genes (Jin et al., 2018a).

### 2.4 Volume-regulated anion channel (VRAC)

VRAC is an important anion channel that regulates cell volume in response to swelling stress, and was initially identified primarily as a way for cells to release Cl^−^ ions or specific organic osmolytes, such as halides or glutamate, followed by osmotic water, during regulated volume reduction (RVD) (Pedersen et al., 2015; Jentsch, 2016). Under these conditions, the Cl^−^ current through the VRAC, named IClswell, displays a slight outward rectification and is independent of voltage activation and time (Schlichter et al., 2011). Recent studies have identified the leucine-rich-repeat-containing 8A (LRRC8A), a hexamer consisting of four transmembrane helices per subunit, was an essential contributor to VRAC current triggering. To show functional gating selectivity and volume sensitivity, LRRC8A was usually found to act in conjunction with at least one other LRRC8 member (LRRC8B-E) (Voss et al., 2014; Gaitan-Penas et al., 2016). A multitude of studies have proposed that LRRC8A is involved in a variety of pathophysiological processes, including cell apoptosis, proliferation, migration, metabolism and secretion, all of which involve changes in local cell volume (Chen et al., 2019a). VRAC facilitate cell survival under hypotonic conditions as detailed above. Increased cell volume has been reported to lead to increased membrane tension, activation of mechanosensitive and Ca^2+^ permeable channels, leading to cell damage and death, and increased deposition of matrix proteins (Warntges et al., 2001). Also, increased proximal tubular hyperosmotic stress responds to mechanical stress and osmotic pressure, rearranging focal adhesions in tubular epithelial cells to induce EMT, which in turn leads to renal fibrosis (Miyano et al., 2021). Regardless of hypo- or hyperosmotic stress conditions, cell volume recovery is critical for homeostasis and renal function of the organism. However, the mechanism between increased cell volume and VRAC activation is not yet fully understood, which may be partly through downstream signaling or sensing changes in intracellular ionic strength (Voets et al., 1999; Syeda et al., 2016). Also, the actin cytoskeleton may be involved in this process. In RVD, the actin cytoskeleton (filaments) depolymerizes during cell swelling, which also stimulates microtubule expression and is involved in VRAC stimulation (Burow et al., 2015). The functional integrity of actin filaments and microtubules is a prerequisite in maintaining the effective RVD responses. Additionally, disruption of the cytoskeleton of glomerular and tubular cells, especially of the actin and microtubule network, is strongly associated with pro-fibrotic effects (Parrish, 2016). Therefore, we hypothesized that VRAC may have an important role in mediating cytoskeletal protein-driven renal fibrosis through regulation of cell volume.

In addition, in the absence of osmotic challenge, other stimuli such as hypoxia, serum, intracellular Ca^2+^, ATP, phospholipids and other intracellular signals have been reported to stimulate VRAC (Patel et al., 1998; Catacuzzeno et al., 2011; Kunzelmann, 2015). Friard et al. (2019) found that TGF-β1 also activated VRAC/LRRC8A channels to trigger chloride currents, but at a relatively slow rate and with weak current amplitude. Using a biochemical or pharmacological approach, they further showed that inhibition of VRAC/LRRC8A attenuated TGF-β1-induced expression of the EMT phenotype and associated markers in human proximal tubular epithelial cells, and that this mechanism may be related to its ability to permeabilize glutathione (GSH) and thus counteract increased intracellular ROS levels (Friard et al., 2019). Studies on the association of volume-sensitive Cl^−^ -channels in renal tubular epithelial cells may provide interesting models for better understanding the process of renal epithelial mesenchymal transition under stress. Furthermore, establishing a new approach based on VRAC/LRRC8A for the treatment of renal fibrosis has great potential, which requires more research.

### 2.5 Voltage-dependent anion channels

The voltage-dependent anion channel (VDAC) is a multifunctional channel that controls cellular energy, metabolism, oxidative stress and apoptosis (Shoshan-Barmatz et al., 2010). They are a class of bidirectional transport porins located in the mitochondrial outer membrane different from classical ion channels. Three types of VDAC (VDAC1-3) have been identified in higher eukaryotes, of which VDAC1 is highly expressed in most cell types. Nowak et al. (2020) recently found that VDAC1 expression was reduced in mitochondria of an ischemia/reperfusion (I/R) kidney injury mouse model compared to WT mice, and that VDAC1 deficiency resulted in reduced mitochondrial respiration and ATP levels with increased mitochondrial fission in non-injured kidneys, supporting the role of VDAC1 in maintaining mitochondrial dynamics and energy metabolism. Importantly, the overall absence of VDAC1 impedes the morphological regeneration of proximal tubules and the recovery of renal function, increases collagen deposition in the post-ischemic kidney and exacerbates interstitial renal fibrosis (Nowak et al., 2020). In contrast, Li et al. (2022b) found that knockdown of VDAC1 significantly attenuated ischemic injury-induced apoptosis and mitochondrial damage in renal tubular cells. This discrepancy may be related to complex mechanisms, differences in disease and cell types. Interestingly, Tf-D-LP4, a peptide targeting VDAC1, was found to be effective in treating nonalcoholic fatty liver disease (NAFLD), reducing inflammation, liver fibrosis, and normalizing liver enzymes (Pittala et al., 2019), and VBIT-4, an inhibitor of VDAC1 oligomerization, attenuates fibrosis caused by increased cardiac aldosterone (Klapper-Goldstein et al., 2020). Thus, VDAC1 appears to be a promising treatment for fibrotic disease. Although the role of VDAC1 in different renal diseases has been established, valuable evidence to support the claim that VDAC perturbation causes or exacerbates renal fibrosis remains to be determined.

### 2.6 Chloride intracellular channels

Chloride intracellular channel 4 (CLIC4) belongs to a highly conserved and most extensively studied member of the CLIC family of proteins (Shukla and Yuspa, 2010). It is present in the inner cell membrane and abundant in the cytoplasm as the soluble form. CLIC4 is involved in various cellular functions, including regulation of cell proliferation, apoptosis, pH homeostasis, cell cycle, angiogenesis and differentiation (Shukla et al., 2009; Shukla and Yuspa, 2010). In the kidney, CLIC4 null mice support vacuolar acidification and are associated with abnormal dilatation of proximal tubules (Ulmasov et al., 2009). Using biochemical and morphological analysis, CLIC4 was reported to be overexpressed in the proximal tubular region of rats with hypertensive nephropathy compared to normal rats (Hatziioanou et al., 2018), inhibition of CLIC4 largely reduced TGF-β1-induced transdifferentiation of fibroblasts from myofibroblasts and α-SMA as well as ECM component expression (Shukla et al., 2014). Indeed, CLIC4 directly enhances TGF-β signaling by binding to Smad2/3 and preventing their dephosphorylation (Shukla et al., 2009). Furthermore, CLIC4 is able to regulate the matrix degradation activity of MMP-14 (Hsu et al., 2019), which has been shown to be a relevant mediator of vascular senescent renal fibrosis (Vasko et al., 2014). We are looking forward to new research in this area as it is an interesting and relatively unexplored target in renal fibrosis.

### 2.7 Claudin proteins-associated channels

The paracellular channel through the tight junctions are an important pathway for transepithelial Cl^−^ transport in the kidney, which provides approximately 70% of Cl^−^ reabsorption (Sansom et al., 1984; Schild et al., 1988). The claudin proteins (CLDNs), forming the channels that connect two extracellular compartments by passing perpendicular to the membrane plane, are the main components of tight junction generation (Hou et al., 2013). Paracellular permeability depend on the complement of CLDNs in each nephron segment, where they are expressed in a nephron-specific manner. The localization of CLDNs in the nephron varies among mammals, with several CLDNs normally expressed in the renal tubule (2, 10a, 17), podocytes (5, 6) and collecting ducts (3, 4, 7 and 8) (Hou et al., 2013). Claudins are reported to be altered during cellular EMT and are involved in the feedback regulation of epithelial cell phenotypic transformation (Quaresma et al., 2020). Dan et al, (2019) showed that CLDN-2 expression was initially increased and then decreased in a mouse obstructive nephropathy model, and silencing of CLDN-2 enhanced Ras homolog family member A (RHOA)-mediated activation of myocardin-related transcription factor (MRTF), a major regulator of EMT, to promote epithelial reprogramming in renal fibrosis. In addition, CLDN5-specific deletion in podocytes significantly exacerbated interstitial renal fibrosis after UUO by blocking WNT inhibitory factor-1 (WIF1) secretion (Sun et al., 2022). Therefore, it is possible that deletion of CLDNs contributes to the pathogenesis of renal fibrosis. See Table 1 for a full listing of ion channels in renal fibrosis.

**TABLE 1:** The expression and function of various ion channels for renal fibrosis.

Target	Model/species	Expression	Treatment	Action/effect	Comments	References
CFTR	UUO mice models; MDCK/HK-2 cells	↓	Gene mutation, Knockdown of CFTR, inh172/GlyH101 (inhibitor)	↑ Interstitial ECM, α-SMA, tubular atrophy, inflammation	Activating Wnt/β catenin pathway	Zhang et al. (2017)
	Type 2 diabetes mice; RTECs	NA	Up-regulation of CFTR by CDX2	↓EMT, ↑cell junction proteins	Interfered with β-catenin activation	Liu et al. (2021)
TMEM -16A	UUO/high-fat diet murine models; HK-2 cells	↑	Knockdown of TMEM16A, CaCCinh-A01 or T16Ainh-A01 (specific inhibitor)	↓EMT, α-SMA, fibronectin, collagen I	Inhibiting Smad2/3 and ERK1/2 phosphorylation	Li et al. (2022a)
	MCT cells	NA	Upregulation of TMEM16A by CLCA1; T16Ainh-A01	↑Matrix protein, SASP; ↓matrix protein, SASP	Drive akt-mTORC1 axis by activating TMEM16A	Lee et al. (2021)
ClC-5	UUO mice models; HK-2 cells	↓	Infected with AdClC-5; ClC-5 -knockout cells	↓EMT, renal atrophy, CTGF, collagen III/IV, Inflammatory; ↑EMT	ClC-5 upregulation inhibits activation of NF-κB/MMP-9 signaling pathway	Yang et al. (2019b)
	ClC-5 knockout mice	NA	High citrate diet	↓Tubular atrophy, interstitial fibrosis, cystic changes vs. zero citrate diet groups	Inhibiting TGF-β1 signaling pathways	Cebotaru et al. (2005)
VRAC	HEK-293/HK-2 cells under TGF-β1	↑	Knockdown of VRAC, DCPIB, 20 μM (VRAC inhibitors)	↓EMT, E-cadherin, GSH, ↑ROS, fibronectin, MMP9, collagen IV	Inhibition of VRAC permeability to GSH→ increased ROS	Friard et al. (2019)
VDAC1	VDAC1-deficient mice; WT mice	↓	Ischemia	↑Extracellular matrix proteins	Increased mitochondrial fission, reduced renal ATP content	Nowak et al. (2020)
Orai1	UUO/high-fat diet murine models; HK-2 cells	↑	Knockdown of Orai1, SKF96365(Orai1 Inhibitors)	↓Fibrotic lesions, EMT, fibronectin, TGF-β1, α-SMA, collagens I/III/IV	Enhancing Ca^2+^ influx, suppressing Smad2/3 phosphorylation	Mai et al. (2016)
	I/R injury recovery rats under high salt diet/angiotensin II; renal CD4 T cells	↑	YM58483/BTP2(SOCE inhibitors)	↓Ca^2+^ influx, Th17 expression, renal function, fibrosis, inflammation	Blocking IL-17 activation	Mehrotra et al. (2019)
P2X7R	UUO mice models	↑	*P2X7R* knockout	↓Collagen, TGF-β, myofibroblasts, tubular atrophy, macrophage infiltration, apoptosis	Inhibited TGF-β signaling pathway and macrophage infiltration	Goncalves et al. (2006)
	Pyelonephritis postrenal scar mice	NA	*P2X7R* knockout brillian blue G (P2X7R antagonist)	↓ Interstitial fibrosis	Inhibited macrophage infiltration	Therkildsen et al. (2019)
	Diabetic mice; Primary human mesangial cells	↑	*P2X7R* knockout AZ11657312(P2X7R inhibitors)	↓Collagen IV, macrophage, MCP-1	Inhibited macrophage infiltration by reducing MCP-1	Menzies et al. (2017)
P2X4R	UUO mice models	↑	*P2X4R* knockout	↑Collagen I, TGF-β, fibronectin, α-SMA	Enhanced TGF-β expression	Kim et al. (2014)
TRPC3	UUO mice models; renal fibroblasts	↑	*TRPC3* knockout, pyr3 10 μM (TRPC3 specific inhibitors), TRPC3 knockdown	↓Fibroblast activation, ECM, myofibroblast differentiation	Inhibited Ca^2+^ influx, ERK1/2 phosphorylation	Saliba et al. (2015)
TRPC6	UUO mice models; HEK293 cells	↑	*Trpc6* knockout, BTP2 (TRPC inhibitors)	↓Collagen-1, CTGF, α-SMA, MMP-2, MMP-9, fibrosis	Protected the kidney through soluble klotho	Wu et al. (2017b)
	UUO murine models; TEC	↑	*Trpc6* knockout	↓EMT, fibrotic injury	Negatively regulates the AKT-mTOR and ERK1/2 signaling pathways	Zhang et al. (2020)
TRPV1	DOCA-salt hypertension mice	NA	TRPV1^⁻/⁻^ mutant	↑glomerulosclerosis, ECM, tubular injury vs. WT mice	Inhibition of TGF-β and its downstream pathways	Wang and Wang, (2011)
TRPV4	DOCA-salt hypertension rats	NA	Dietary apigenin (TRPV4 activator)	↓Extracellular matrix proteins	Ca^2+^ influx→ activated AMPK/SIRT1→inhibited TGF-β1/Smad2/3 signaling pathway	Wei et al. (2017)
TRPM7	UUO murine; normal rat kidney fibroblast cell and epithelial cell	↑	NS8593 (TRPM7 inhibitor)	↓Collagen I, kidney atrophy, α-SMA, cell proliferation fibronectin	Inhibited TGF-β1/Smad signaling pathway	Suzuki et al. (2020)
ENaC	*Nedd4-2* ^−/−^ mice	↑	Amiloride (ENaC specific inhibitor)	↓Na^+^ reabsorption, fibrosis, Kim-1	Reduced Na^+^ reabsorption, aldosterone, hypertension	Henshall et al. (2017)
NKA	Porcine proximal tubular cell (LLC-PK1)	NA	Marinobufagenin (NKA activator)	↑EMT	Increased the protein levels and nuclear localization of Snail	Fedorova et al. (2009)
	UUO mice	↓	pNaKtide (NKA mimic)	↓ TGF-β1, ECM, myofibroblasts, ROS	Inhibited Src and its downstream effector	Cheng et al. (2019)
	5/6 nephrectomy mice	NA	DRm217(NKA specific antibody)	↓Renal tubular cells apoptosis, interstitial injury, renal fibrosis	Blocked Src activation	Wang et al. (2019b)
NHE1	Rat mesangial cells under aldosterone	↑	Knockdown of NHE1 by shRNA	↓Fibronectin	Stimulated NHE1 *via* the ERK1/2 pathway	Zhang et al. (2010)
	Nephrectomy-induced CKD mice; Rat renal tubular cells (NRK-52E)	NA	Fucoxanthin (NHE1 activator)	↓Fibronectin, collagen, cell apoptosis	Upregulation of NHE1 *via* the PPARα pathway	Chen et al. (2018)
K_Ca_3.1	UUO mice model; Mouse kidney fibroblasts	↑	*K*_*Ca*_ *3.1* Knockout TRAM-34(K_Ca_3.1 selective inhibitor)	↓Fibroblasts proliferation, collagen I/III, α- SMA	Inhibited fibroblast proliferation by cell cycle arrest in G0-G1 phase	Grgic et al. (2009a)
	Diabetic mice; Human renal interstitial fibroblasts	NA	*K*_*Ca*_ *3.1* Knockout, TRAM-34	↓ECM, fibroblasts activation, MMP2, MMP9	Inhibited activation of fibroblasts and phosphorylation of Smad2/3 and ERK1/2	Huang et al. (2014a)
K_Ca_1.1	UUO and folic acid mice models; HK-2, NRK-49F, NRK52E cells	↓	*K*_*Ca*_ *1.1* knockout; NS1619**/**BMS191011 (channel openers)	↑Fibronectin, α- SMA, collagen III/I; ↓fibrosis, fibronectin, α-SMA, p-Smad2 and p-Smad3	K_Ca_1.1 activation accelerates TGF-β receptor degradation through caveolae pathway	Wang et al. (2022)
Kv1.3	5/6 nephrectomy rat	↑	Margatoxin (selective Kv1.3 inhibitor)	↓Leukocytes, collagen III, cell cycle marker, Cdk4	Inhibited cell cycling, cellular proliferation	Kazama et al. (2012)
	UUO rat	↑	Margatoxin	↓Leukocytes, α- SMA, myofibroblast	Inhibited leukocytes proliferation	Abe et al. (2019)
K_ATP_	Spontaneously hypertensive rats	↓	Iptakalim (sensitive K_ATP_ opener)	↓Blood pressure, proteinuria, collagen IV, fibronectin, MMP9	Inhibited endothelin 1 and TGF-β1	Xue et al. (2005)

Abbreviations: ↓, Decreased; ↑, Increased; UUO, unilateral ureteral occlusion; MDCK, renal distal tubular Madin–Darby canine kidney cells; HK-2, human proximal tubule cells; RTECs, normal rat kidney tubular epithelial cells; CDX2, caudal-type homeobox transcription factor 2; MCT, murine proximal tubular epithelial cells; CTGF, connective tissue growth factor; HEK, human Embryonic Kidney; AMPK, AMP-activated protein kinase; CLCA1, chloride channel accessory 1; SASP, senescence associated secretory phenotype.

## 3 Ca^2+^ channels

Ca^2+^ signaling is a key determinant of homeostasis and cellular function. As a ubiquitous and essential second messenger, the cytosolic Ca^2+^ is involved in the regulation of various cellular functions, such as cell activation, proliferation, development, differentiation, survival, homeostasis and effector functions (Berridge, 2016). In the kidney, Ca^2+^ is also involved in the endocrine regulation of renal blood flow, glomerular filtration, and tubular handling of water and electrolytes. Dysregulation of Ca^2+^ signaling is often thought to play an important role in the development of renal diseases such as polycystic kidney, acute kidney injury, glomerular disease, and diabetic nephropathy (Yang and Yang, 2013; Ning et al., 2021). In addition, critical regulation of intracellular Ca^2+^ levels during driving EMT is necessary for the translation of extracellular signals into gene expression effects and execution of cellular behavior. In this scenario, Ca^2+^-permeable ion channels that regulate Ca^2+^ signaling can have a dramatic impact on cellular phenotypes. This review will discuss the role of Ca^2+^-release-activated Ca^2+^ channels (CRACs), purinergic P2 receptors, and transient receptor potential (TRP) channels in the process of renal fibrosis.

### 3.1 Ca^2+^ release-activated Ca^2+^ channels

**FIGURE 2:**
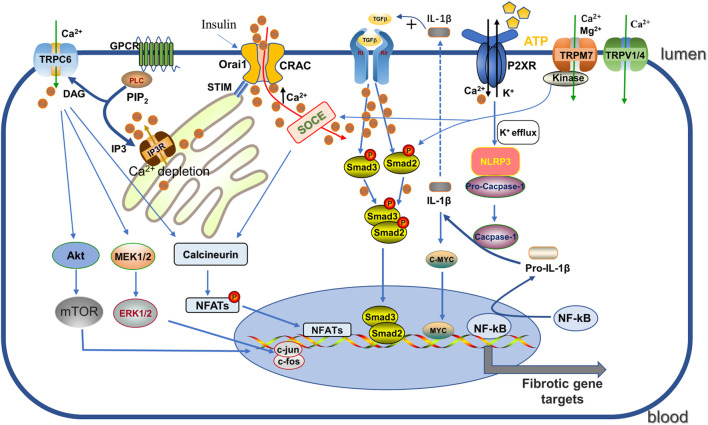
Regulation of CRAC, P2XR, TRP channels. (1) GPCR activates PLC, then further decompose the phosphorylated PIP2 into DAG and IP3. IP3 acts on IP3R on the ER, triggering Ca^2+^ release leading to depletion, which is sensed by STIM and then binds and activates the ORAI pore-forming channel CRAC, facilitating Ca^2+^ entry. Enhanced Ca^2+^ promotes the phosphorylation of Smad2/3 to enhance the TGF-β1 signaling pathway, and binds to calcineurin and promotes NFAT activity. (2) Activated P2X7R induces NLRP3 inflammasome activation by K^+^ efflux and catalyzes pro-IL -1β cleavage to IL-1β, which not only promotes the TGF signaling pathway but also enhances c-myc transcriptional enhancement of fibrosis. (3) TRPC6 promotes the fibrotic process by enhancing AKT-mTOR and ERK1/2 pathways as well as the calcineurin/NFAT pathway. DAG: diacylglycerol; GPCR: G protein coupled receptor; IP3: 1, 4, 5-triphosphoinositol; IP3R: inositol 1,4,5-triphate receptor; PIP2: phosphorylated phosphatidylinositol 4, 5-diphosphate; PLC: phospholipase C; NFATs: nuclear factor of activated T cells; IL-1β: Interleukin-1β; NLRP3: nucleotide-binding oligomerization domain-like receptor family pyrin domain-containing-3.

The influx of extracellular Ca^2+^ or the release of intracellular Ca^2+^ stores (about 90% stored in the endoplasmic reticulum (ER) and mitochondria) is the main source of increased cytoplasmic Ca^2+^ levels (Prakriya and Lewis, 2015). As a major pathway mediating critical Ca^2+^ entry in several non-excitable and excitable cells, store-operated Ca^2+^ entry (SOCE), triggered by Ca^2+^-dependent depletion in the ER through the store-operated Ca^2+^ (SOC) channel (mechanistically we also call Ca^2+^ release-activated Ca^2+^ channel (CRAC), has been explored in detail. In this process, two highly conserved molecular proteins play a decisive role: one is stromal interaction molecule (STIM) responsible for sensing ER Ca^2+^, including STIM1 and STIM2, and another is Orai, the store-operated channel protein, divided into three subtypes, Orai1 to Orai3, which are all important components of SOCE. Specifically, when the inositol 1,4,5-trisphosphate receptor (IP3) receptor is activated, the intracellular space in the ER is depleted by Ca^2+^ release, which is sensed by STIM1 and subsequently relocated by binding to the microtubule plus-end binding protein EB1 (shifted to the Orai1 protein in the plasma membrane), activating CRAC and thus facilitating Ca^2+^ influx (Prakriya and Lewis, 2015; Chen et al., 2019b). SOCE plays an important role in regulating cell migration, proliferation, apoptosis, gene regulation, and secretion (Kim et al., 2011) (see Figure 2).

STIM/Orai-dependent SOCE is closely associated with the progression of renal fibrosis. It was shown that Orai1 expression was upregulated in the kidneys of UUO and high-fat diet (HDF)-induced renal fibrosis mouse models and in renal tubular epithelial cells from kidney biopsies of patients with fibrotic nephropathy, such as focal proliferative sclerosis and tubulointerstitial nephritis (Mai et al., 2016). Investigations in HK2 cells revealed that TGF-β1-driven EMT as well as fibronectin and α-SMA expression were significantly reduced by Orai1 silencing that was attributed to the prevention of abnormal Ca^2+^ influx and inhibition of Smad2/3 phosphorylation (Mai et al., 2016; Ma et al., 2018). The renoprotective effect of Orai1 deficiency was similarly confirmed *in vitro* experiments in UUO mice and high-fat fed ApoE^−/−^ mice (Mai et al., 2016). Studies on podocytes from diabetic nephropathy mice suggest that specific Orai1 deletion prevents insulin-stimulated SOCE, slit-diaphragm disruption and proteinuria, possibly due to chronic stimulation of Orai1 activation or aberrant Ca^2+^ signaling, which in turn activates Ca^2+^-regulated phosphatases leading to remodeling of the actin cytoskeleton (Kim et al., 2021). Furthermore, using siRNA to down-regulate STIM1 expression in cultured podocytes from diabetic nephropathy rats serum could reverse the decrease in autophagy and inhibit EMT by restoring Ca^2+^ homeostasis (Jin et al., 2018b; Jin et al., 2019). Spontaneously hypertensive rats with truncated STIM1 exhibit albuminuria, glomerular and interstitial fibrosis (Dhande et al., 2020). Notably, in cultured human mesangial cells, activation of glucagon-like peptide-1 receptor (GLP-1R) or thapsigargin on SOCE inhibited high glucose- and TGF-β1-stimulated matrix protein synthesis (Wu et al., 2015; Huang et al., 2019). In contrast, knockdown of Orai1 using targeted nanoparticle siRNA delivery significantly increased fibronectin and type IV collagen expression in mesangial cells as well as mesangial expansion by a mechanism running through inhibition of pre-fibrotic Smad1 and Smad3 phosphate activation (Wu et al., 2017a; Chaudhari et al., 2017). This suggests that SOCE appears to have complex functions in mesangial cells, with different functions in response to high glucose or high fat stimuli in different cell types and technical approaches. Collectively, these results support that STIM/Orai-dependent SOCE may be an important therapeutic approach to study the progression of renal fibrosis.

Strong T helper 17 (Th17) cells inflammatory response exacerbates ischemia-reperfusion-induced acute kidney injury (AKI) and mediates inflammatory cell infiltration promoting renal fibrosis (Peng et al., 2015; Mehrotra et al., 2017). In studies of Th17 differentiation in AKI rats, Mehrotra et al. (2019) revealed the important role of orai1-dependent SOCE involved in Th17 differentiation and inflammatory responses during AKI and AKI to CKD transformation. This was manifested as Orai1 mutation leading to Th17 cell damage, and almost no IL-17 was expressed in Oria1^−/−^ cells, whereas stimulated IL-17 expression was inhibited by SOCE antagonist. In addition, Orai1 was consistently expressed in CD4 T cells after recovery from AKI and the use of the SOCE pathway inhibitor (YM58483/BPT2) significantly attenuated ischemia-reperfusion kidney injury, which was associated with reduced Th17 cells. Similarly, YM58483/BPT2 attenuated the progression of inflammation, proteinuria and interstitial fibrosis induced by exposure to angiotensin II or high salt diet I/R recovery in mice, which is thought to be reactivated by Th17 cells (Mehrotra et al., 2019). Thus, continued Orai1 expression may underlie the susceptible activation of Th17 cells, suggesting that Orai-mediated Ca^2+^ mechanisms may be an attractive therapeutic target against CKD progression or immune-mediated inflammatory renal fibrosis.

### 3.2 Rurinergic P2 receptors

Adenosine 5′-triphosphate (ATP) is not only an important intracellular energy carrier, but its extracellular release also has a role as a signaling molecule (Linden et al., 2019), for example, as a damage-associated molecular pattern (DAMP) signal involved in the inflammatory response to kidney tissue injury (Arulkumaran et al., 2013). ATP signaling acts through purinergic P2 receptors, including metabolic P2Y and ionotropic P2X, to participate in intracellular Ca^2+^ regulation, fluid secretion, glomerular filtration rate and epithelial transport (Ralevic and Burnstock, 1998; Vallon et al., 2020). P2Y receptors have eight subtypes (P2Y1, 2, 4, 6 and 11–14) and act as a class of G protein-coupled receptors that initiate second messenger cascade signaling by binding ATP, thereby increasing intracellular Ca^2+^ release (Geyti et al., 2008), and the P2X receptor family has seven subtypes (P2X1∼7), which are widely distributed in various cell types and form permeable Ca^2+^ non-selective cation channels. In renal tubular epithelial cells, stimuli triggered by a range of factors including cellular mechanical stretch (e.g., increased sensory blood flow and tubular fluid flow), pathogen invasion, cell injury, or agonist binding can induce the release of ATP into the extracellular space, activating the P2X receptor and allowing the influx of Ca^2+^, Na^+^, and other cations, thereby triggering biological effects (Monaghan et al., 2021). P2X7R and P2X4R are key subtypes in the pathophysiology of the kidney, while they share some commonalities including structural commonalities and physical and functional interactions but may even have opposite properties (Craigie et al., 2013).

P2X7R was initially identified in macrophages and monocytes and plays an important role in the regulation of pro-inflammation and pro-apoptosis (Arulkumaran et al., 2011). Persistent interstitial inflammation drives worsening kidney injury, fibrosis and functional impairment. P2X7R is involved in the rapid processing and release of cytokines IL-1β and IL-18 and promotes apoptosis and necrosis, which is associated with the activation of the nucleotide-binding oligomerization domain-like receptor family pyrin domain-containing-3 (NLRP3) inflammasome (Baron et al., 2015). Recently, it has been observed that P2X7R serves as a possible target with potential pro-fibrotic function in several types of fibrotic diseases (Gentile et al., 2015). The distribution of transient expression of P2X7R was identified in fibrotic kidneys of UUO-induced mouse model (Goncalves et al., 2006), whereas P2X7R is usually expressed at low levels in normal kidneys (Turner et al., 2003). Using genetic tools, P2X7 knockout mice showed significantly reduced renal interstitial macrophage infiltration and myofibroblast numbers as well as TGF-β1 expression compared to wild-type mice in a UUO model (Goncalves et al., 2006). Consistent with this finding, Therkildsen et al. studied sporadic renal cortical fibrosis in mice exposed to *E. coli* producing the virulence factor alpha-hemolysin (HlyA), and the functional significance of P2X7R was demonstrated by using the most potent antagonist, brillian blue G, which significantly reduced fibrosis and macrophage infiltration in mice with renal scarring caused by pyelonephritis (Therkildsen et al., 2019). These results suggest that deletion of P2X7R effectively inhibit renal fibrosis after obstruction and infection, and macrophage infiltration seems to be an important part of the process.

In diabetic patients, extensive renal expression of P2X7R is associated with severe mesangial expansion and fibrosis (Menzies et al., 2017). P2X7R activation is involved in TGF-β secretion and ECM production in mesangial cells (Solini et al., 2005), in turn P2X7R deficiency prevents glomerular macrophage accumulation and collagen IV deposition (Menzies et al., 2017). Long-term high-fat feeding of mice resulted in renal inflammation and oxidative stress as well as alterations in renal structure, which were associated with NLRP3 inflammasome activation (Stienstra et al., 2011). It was found that P2X7R activation, NLRP3 inflammasome formation, caspase-1 induction, pro-IL-1β, pro-IL-18 activation all increased after “metabolic” renal injury and were all inhibited by P2X7R silencing (Solini et al., 2013). IL-1β activates autophagic flux to promote transcription factor MYC accumulation, and up-regulation of MYC target genes are essential for driving progressive renal tubulointerstitial fibrosis (Lemos et al., 2018). Therefore, blocking the axis of P2X7R-NLRP3 inflammasome may be an important target to protect kidney function from fibrosis progression. This mechanism has also been validated in the UUO model (Nam et al., 2019) (see Figure 2). Interstitial fibroblasts in early kidney injury contribute to kidney repair. After acute kidney injury, ATP released from necrotic tubular cells rapidly acts on adjacent interstitial fibroblasts by binding to P2X7R, inducing cell death (Ponnusamy et al., 2011). P2X7 receptor blockade not only reduces interstitial fibroblast loss but, interestingly, also promotes renal recovery by reducing the infiltration of innate and adaptive effector cells, increasing the infiltration of regulatory T cells (Tregs) during recovery, and delaying renal fibrosis and scar formation (Ponnusamy et al., 2011; Koo et al., 2017). Thus, these reports confirm that P2X7R plays a key role in the development and progression of renal fibrosis, providing a critical target and direction for the treatment of renal fibrosis.

In contrast to P2X7R, P2X4R has a nephroprotective effect in the UUO mouse model (Kim et al., 2014). Vallon et al. (2020) speculated that the reason may be related to the fact that P2X4R controls T cell migration and induces its toxic function subsequently promoting fibroblast apoptosis. However, the role of P2X4R in fibrosis progression needs to be carefully understood, as ATP-P2X4 signaling can likewise induce activation of the NLRP3 inflammasome, exacerbating the progression of renal tubulointerstitial inflammation and fibrosis (Chen et al., 2013; Han et al., 2020).

### 3.3 TRP channels

Transient receptor potential (TRP) channels are a large family of Ca^2+^ permeable channels that are widely expressed in a variety of cells types. Since the original TRP genes appeared in *Drosophila*, at least 27 TRP genes have been identified in mammals (Wu et al., 2010). As an important pathway for Ca^2+^ to flow into the cytoplasm, TRP channels regulate Ca^2+^ to depolarize cell membranes, change enzyme activities, and achieve signal cascade response (Zheng, 2013). Based on differences in their amino acid sequences and topologies, TRP channels can be divided into seven subfamilies (canonical TRPC, vanilloid TRPV, melastatin TRPM, polycystin TRPP, ankyrin TRPA, mucolipin TRPML, and NompC-like TRP) (Nelson et al., 2011). Although TRP channels belong to the non-voltage gated Ca^2+^ superfamily, they share similarities in structural features, including six transmembrane segments (S1-S6) as well as intracellular NH2 and COOH terminations and a pore lining (composed of S5 and S6), which subunits complement each other to form a tetramer (Hardie, 2011). As an integral membrane protein of cells, TRP channels integrate multiple stimuli including temperature, smell, pH, pressure, mechanical stimuli, chemical reagents, herbs as well as poisons and respond to intracellular Ca^2+^ signaling, participating in a variety of pathophysiological processes (Vriens et al., 2008; Moran et al., 2011). Several studies have linked different fibrotic disease types, including renal fibrosis, to TRP channels (Inoue et al., 2019; Okada et al., 2021). Here, we will describe a few typical examples of the role of TRP channels in renal fibrosis, due to the large size of the TRP channel family and limited space.

TRPC3/6/7 is a constitutively active receptor-operated channel that can be regulated by various signaling molecules such as phosphatidylinositol 4,5-bisphosphate (PIP2), diacylglycerol (DAG), ATP, calmodulin and reactive oxygen species (Lemonnier et al., 2008). These TRPC proteins can be activated by stretching under mechanical stress and amplify downstream cellular signals coupled by the integrated stimulus *via* calcium permeation and membrane depolarization (Scott et al., 2006). The potential role of TRPC3 and TRPC6 in renal fibrosis has been studied in an animal model of UUO induction, and both of them are upregulated in the obstructed kidney (Saliba et al., 2015; Wu et al., 2017b). TRPC3 channels, accompanied by direct Ca^2+^ influx, have been shown to promote renal fibroblast proliferation, myofibroblast differentiation, and extracellular matrix remodeling upon the DAG and the DAG generating angiotensin II (Ang II), which was notably reduced by the TRPC3 channels inhibitor pyr3 or siRNA knockdown (Saliba et al., 2015). The mechanism underlying increased interstitial fibroblasts proliferation and inflammation may involve Ca^2+^ entry *via* TRPC3, which activates and phosphorylates extracellular signal-regulated kinase (ERK1/2), a major regulator of the cell cycle (Saliba et al., 2015). TRPC3^−/−^ mice exhibit diminished renal injury, inflammation, and protection against UUO-induced renal fibrosis (Saliba et al., 2015). The contribution of TRPC3 to the fibroblast fibrogenic response may also be related to its physical interaction with NADPH oxidase 2 (NOX2), a membrane-bound reactive oxygen species (ROS)-producing enzyme (Numaga-Tomita et al., 2017).

TRPC6, as described above, is considered to be another key TRPC channel subtype in the progression of renal fibrosis. The functional significance of TRPC6 channels in pathogenesis of renal diseases, including focal segmental glomerulosclerosis (FSGS), diabetic nephropathy, immune-related nephropathy and chronic kidney disease was extensively studied [more details see the review (Hall et al., 2019)]. TRPC6 expression is significantly increased in UUO induced fibrotic mice as compared to normal renal tissue (Wu et al., 2017b), and also activated under ROS produced by NADPH oxidase (NOX) as TRPC3 (Kim et al., 2013; Ilatovskaya et al., 2018). Directly tested for its involvement in the activation of EMT by AKT-mTOR and ERK1/2 pathways in UUO mouse kidneys, BTP2 (a nonselective TRPC6 inhibitor) or TRPC6 knockdown was found to blunt this effect (Kong et al., 2019; Zhang et al., 2020) (Figure 2). Blockade of TRPC6 with another selective inhibitor BI-749327 observed similar protection in obstructed kidneys (Lin et al., 2019). Indeed, TRPC6-dependent elevation of cellular solute Ca^2+^ activates calcineurin, which stimulates the nuclear factor of activated T cells (NFAT) transcriptional pathway to induce fibroblast differentiation (Davis et al., 2012; Lin et al., 2019; Gu et al., 2020). Studies have demonstrated that TRPC6 and NFAT form a mutual positive feedback loop that aggravates the renal fibrosis (Nijenhuis et al., 2011). TRPC6 channel also affects components of the innate immune response. It has been reported that TRPC6 channels regulate CXC chemokine receptor 2 (CXCR2)-related chemotaxis by mediating Ca^2+^ influx (Lindemann et al., 2013), promoting renal tubular cell senescence and renal fibrosis by inducing mitochondrial dysfunction (Meng et al., 2022). Interestingly, Klotho, a single-channel type 1 transmembrane protein with anti-renal fibrotic effects, had no effect on obstruction-induced fibrosis in TRPC6 knockout mice, suggesting that the renal protective effect of Klotho in UUO is partly mediated through inhibition of TRPC6 (Satoh et al., 2012; Wu et al., 2017b). In diabetic nephropathy, excessive activation of TRPC6 channel activity plays an important role in podocyte apoptotic injury (Staruschenko et al., 2019), and Wnt/β-catenin signaling pathway may be active in this process (Li et al., 2013). Notably, TRPC6 knockdown had no effect on tubulointerstitial inflammation and fibrosis in autoimmune glomerulonephritis and aging rats, although it significantly reduced glomerular sclerosis (Kim et al., 2019; Kim and Dryer, 2021), indicating that targeting TRPC6 may have disease specificity in the treatment of glomerular diseases. Overall, the understanding of TRPC3 and TRPC6 in renal fibrosis pathogenesis has expanded in recent years and may facilitate the development of emerging therapeutic strategies.

The TRPV1 channel subtype is a polymodal cation channel involved in cellular environmental crosstalk and has emerged as an important player in the regulation of related diseases such as inflammation, cancer and immune diseases through the integration of physical or chemical stimuli (Bujak et al., 2019). TRPV1 is abundantly expressed in renal tissues, especially in the renal pelvis, and is involved in the regulation of renal hemodynamics and excretory function (Feng et al., 2008; Li and Wang, 2008). Capsaicin activation of TRPV1 greatly alleviated renal fibrosis in UUO and hyperadenine-fed mouse models by reducing myofibroblast activation and preventing phenotypic alterations in renal tubular epithelial cells, and this mechanism is associated with inhibition of TGF-β1-Smad2/3 signaling (Liu et al., 2022). In a mouse model of deoxycorticosterone acetate (DOCA) -salt induced hypertension, TRPV1 knockdown exaggerated renal injury including renal cortical tubulointerstitial injury, fibrosis, and macrophage infiltration, accompanied by increased ECM protein and activation of TGF-β signaling pathway (Wang et al., 2008; Wang and Wang, 2011). In addition, the nephroprotective effect of TRPV1 activation has also been demonstrated in models of diabetic nephropathy and ischemic renal injury (Chen et al., 2014; Wei et al., 2020). Interestingly, TRPV1 knockout mice exhibit a younger metabolism as well as a longer lifespan, predicting that TRPV1 is associated with metabolic disorders, obesity, and aging (Riera et al., 2014).

The vanilloid TRPV4 channel appears to act differently in different models of fibrosis. Increasing evidence suggests that key vanilloid TRPV4 channel subtypes regulate myofibroblast differentiation in cardiac fibrosis, pulmonary fibrosis by integrating mechanical and soluble signals from ECM stiffness and TGF-β1 (Adapala et al., 2013; Rahaman et al., 2014). However, in hypertensive kidney, Wei et al. found that TRPV4-dependent rise of cytosolic Ca^2+^ activated by apigenin triggered AMP-activated protein kinase (AMPK)/sirtuin 1 (SIRT1) pathway and further inhibited TGF-β1/Smad2/3 signaling pathway and extracellular protein expression in renal mesangial and tubular epithelial cells. TRPV4 knockdown abolished this beneficial effect of hypertension-induced renal fibrosis (Wei et al., 2017). The underlying mechanism between TRPV4 and renal fibrosis still needs further elaboration.

The melastatin TRPM7 channel subtype is a bifunctional protein comprised of a cation channel segment linked to an α-type protein kinase domain (Takezawa et al., 2004). TRPM7 promotes SOCE enhancement through its kinase function and increases Ca^2+^-dependent pro-inflammatory and pro-proliferative cytokine effects (Schilling et al., 2014; Faouzi et al., 2017). TRPM7 is significantly upregulated in UUO kidneys compared to normal mice (Suzuki et al., 2020), and its upregulation reportedly contributes to fibrosis (Xu et al., 2015a). Suzuki et al. (2020) showed that inhibition of TRPM7 using NS8593, a small conductance K^+^ channel inhibitor, directly blocked TRPM7 currents, reduced UUO kidney injury, and attenuated renal fibrosis and atrophy. Also, the TGF-β1/Smad signaling pathway was inhibited in this process. Indeed, Smad2 is a substrate of TRPM7 kinase, and inhibition of TRPM7 reduces the expression of TGF-β1/Smad signaling pathway, which contributes to the reduction of renal interstitial and tubular epithelial cell proliferation and ECM production. TRPM7 channel activity is critical for intracellular Mg^2+^ homeostasis (Ryazanova et al., 2010), and alterations in Mg^2+^ metabolism may be associated with TRPM7 downregulation (Sontia et al., 2008). Aldosterone mediates blood pressure-independent renal fibrosis and inflammation *via* Mg^2+^-sensitive pathways (Sontia et al., 2008), suggesting that TRPM7 is involved in the pathogenesis of aldosterone-associated renal fibrosis. However, additional fundamental research is needed to identify specific mechanisms of TRPM7 and Mg^2+^ in renal fibrosis.

## 4 Na^+^ transport

Na^+^ is an important extracellular cation that maintains cellular excitability and is involved in the regulation of water-electrolyte homeostasis, acid-base balance, vascular, neurological, and secretory functions (Greger, 2000). Renal Na^+^ reabsorption is a precisely regulated and controlled process by multiple physiological mechanisms, and has recently received widespread attention. In particular, mechanistic studies of sodium-glucose co-transport have been used as a promising approach for treatment in diabetic nephropathy, and in addition, Na^+^ transport is an important pathological mechanism in salt-sensitive hypertension and cystic kidney fibrosis. Na^+^ channels are usually altered under pathological regulation, for example, TGF-β1-induced EMT is accompanied by an increase in intracellular Na^+^ and water content, suggesting that Na^+^ channels may be relevant in mediating the EMT process (Lamouille and Derynck, 2007; Rajasekaran et al., 2010). Here, we focus on the role of the epithelial sodium channel (ENaC), Na^+^, K^+^-ATPase (NKA), and Na^+^-H^+^ exchangers (NHE) in renal fibrosis.

### 4.1 ENaC

ENaC is a non-voltage gated amiloride-sensitive ion channel and is involved in maintenance of Na^+^ homeostasis and fluid balance by controlling Na^+^ transport in the extracellular fluid of the lumen to epithelial cell (Rotin and Staub, 2021). ENaC is typically composed of structurally similar α, β and γ subunits and formed a heterotrimer expressed in several epithelia, including those of the renal collecting duct, urinary bladder, distal colon, and lung (Hanukoglu and Hanukoglu, 2016). In addition to the regulation of aldosterone sensitivity, extracellular Na^+^, proteases, lipids, angiotensin and other factors also regulate ENaC activity through complex mechanisms (Rotin and Staub, 2021). Chronic ENaC dysregulation has been shown to be the perfect culprit for salt-sensitive hypertension in humans, given its power to accelerate Na^+^-induced damage and water movement through the plasma membrane (Mutchler et al., 2021). However. the functional significance of ENaC channels in renal fibrosis independent of hypertension has been less studied.

In pulmonary and cardiac fibrotic diseases, increased ENaC activity has been shown to be an important mechanistic participant (Jia et al., 2018; Duerr et al., 2020). Increased Na^+^ flux uptake in response to ENaC activation promotes skin fibroblast activation and collagen marker synthesis through the PI3K/Akt signaling pathway, which can be reduced by ENaC blockade (Xu et al., 2015b). However, the deletion of ENaC subunit reportedly causes decreased myogenic self-regulation, leading to renal inflammation and injury with elevated TGF-β1 and collagen III (Drummond et al., 2011), and in turn increased TGF-β1 can reduce ENaC functional activity in epithelial cells (Chang et al., 2008), implying that ENaC decreases in a positive feedback manner in response to renal injury. Li et al. (2007) previously showed that the expression of α, β and γ-ENaC is significantly decreased in the kidney of ureteral obstruction rats when compared to normal tissues, and these changes may lead to the dysfunction of water and Na^+^ metabolism partly through upregulation of cyclooxygenase-2 (COX-2) signaling (Norregaard et al., 2005). Using the same UUO rat model in another study, Yang et al. demonstrated that breviscapine (a flavonoid derived from the herb) prevented the downregulation of γ-ENaC in the obstructed kidney induced by UUO, and significantly reduced the response to renal tubular interstitial fibrosis (Mei et al., 2016). To date, the exact mechanisms of ENaC regulation and release in renal fibrosis remain uncertain, and there are numerous controversies, particularly regarding ENaC expression in different fibrotic disease states and at different stages of fibrosis development.

**FIGURE 3:**
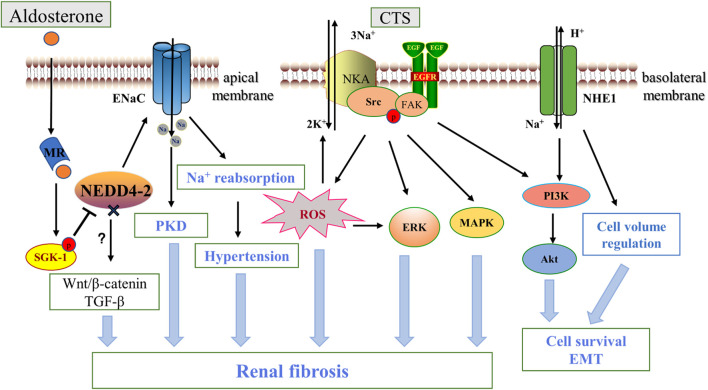
ENaC, NKA, NHE1 and renal fibrosis. Aldosterone binds to the intracellular salt corticosteroid receptor (MR), induces the expression of Na^+^ transport regulators such as SGK1, and phosphorylation inhibits NEDD4-2, which ubiquitinates and degrades ENaC. Increased ENaC activity contributes to PKD disease progression and affects blood pressure through Na^+^ reabsorption, and NEDD4-2 deficiency also promotes fibrosis *via* the Wnt/β-catenin and TGF-β pathways. Na^+^, K^+^-ATPase acts as a scaffolding protein that interacts with CTS to activate Src and then trans-activates EGFR involving a cascade reaction leading to reactive oxygen species generation, which leads to downstream activation of ERK, AKT, MAPK in addition to further activation of NKA. NHE1 promotes renal tubule survival through PI3K/Akt and regulation of cell volume. In addition, ENaC is located in the apical membrane, while NKA and NHE -1 are mainly located in the basolateral membrane. CTS, cardiotonic steroids; PKD, polycystic kidney disease; EGFR, epidermal growth factor receptor; FAK, focal adhesion kinase; FAK, focal adhesion kinase; ERK, extracellular-signal-regulated kinase; MAPK, mitogen-activated protein kinase; PI3K, Phosphatidylinositol 3-kinase.

ENaC on the plasma membrane of the distal nephron, as mentioned above, is normally regulated by various hormones such as aldosterone. As an important regulator of salt reabsorption by mineralocorticoids, serum- and glucocorticoid-inducible protein kinase1(SGK1) triggers a cascade reaction that phosphorylate NEDD4-2, a Nedd4 family ubiquitin protein ligase, to interacts with ENaC *via* the carboxy-terminal Pro-Tyr motif on the channel subunit. NEDD4-2 deletion prevents this interaction and serves as a ligand for increased ENaC activity (Harvey et al., 2001). For example, in ADPKD, increased apical ENaC expression and enhanced Na^+^ reabsorption due to mislocalization of NEDD4-2 are important pathogenic mechanisms (Kaimori et al., 2017). Henshall et al. (2017) showed that renal-tubule-specific NEDD4-2-deficient mice, accompanied by increased Na^+^ reabsorption, resulted in significant fibrotic kidney injury, inflammation and apoptosis of renal tubular epithelial cells as compared to normal mice, and using amiloride, a specific inhibitor of ENaC, inhibited ENaC activity and reduced the extent of kidney lesions. In addition, NEDD4-2-deficient mice are sensitive to dietary Na^+^ due to dysregulation of ENaC (Manning et al., 2020), which further drives the progression of renal interstitial injury and fibrosis through activation of Wnt/β-catenin and TGF-β signaling under a high-Na^+^ diet, and interestingly low Na^+^ diets can rescue this effect, suggesting an important role of ENaC-regulated Na^+^ homeostasis in renal fibrosis (Manning et al., 2021) (Figure 3). High levels of Na^+^ also affect vascular tissue remodeling and fibrosis by impairing phenotypic changes in endothelial cells. Studies have shown that amiloride inhibition of ENaC improves endothelial function, reduces cortical stiffness, and significantly reduces arterial fibrosis and sclerosis in mice (Martinez-Lemus et al., 2017). Moreover, ENaCα subunit deficiency in endothelial cells protects the kidney from ischemic injury by promoting eNOS activation, increasing dependent NO production, and renal perfusion (Tarjus et al., 2019). However, the mechanism by which this elevated Na^+^ reabsorption by ENaC in renal tubules and endothelial cells causes kidney injury and fibrosis remain to be fully understood.

Furthermore, ENaC dysfunction can promote renal fibrosis by facilitating the development of polycystic kidney disease (PKD), and the mechanism may be related to the elimination of antisecretory absorption and purinergic signaling regulation (Pavlov et al., 2015; Sudarikova et al., 2021). Although inhibition of ENaC such as amiloride is not a routine treatment for hypertension because of its lower efficacy compared to other diuretics, the refracted regulatory mechanism of ENaC in fibrosis cannot be ignored and may become a new tool in the treatment of renal fibrosis.

### 4.2 Na^+^, K^+^-ATPase

Na^+^, K^+^-ATPase (NKA) is a cell membrane P-type active cationic hetero-oligomer composed of three subunits, the catalytic α-subunit, the glycosylated β-subunit and the γ-subunit (Feraille and Dizin, 2016). NKA is located primarily in the basolateral membrane and provides the driving force for Na^+^ reabsorption in the apical renal epithelium. ATP-dependent transport of one pump enables three Na^+^ outputs in exchange for two K^+^ entering the cell (Dyla et al., 2020). In addition to acting as an ion transporter protein to maintain cellular ion homeostasis, NKA is also involved in other important cellular processes such as signaling through protein interactions and cell adhesion.

Long-term and systematic studies emphasized the importance of NKA in the pathology of renal fibrosis. Firstly, NKA activity and expression were significantly reduced in renal fibrous tissue from patients with diabetic nephropathy and in the kidneys of acute UUO-induced animal models (Li et al., 2003; Rajasekaran et al., 2010). The cardiotonic steroid (CTS) marinobufagenin (MBG) stimulates NKA signaling has many adverse pathological effects on kidney disease, such as upregulating the expression of the EMT-related transcription factor Snail (Fedorova et al., 2009). Elkareh et al. (2007) also found that MBG stimulates fibroblast collagen synthesis and lead to fibrosis in renal and cardiovascular tissues, which appears to be associated with amplification of ROS production in feed-forward mechanisms *via* activation of Src-EGFR (Yan et al., 2013). Passive immunization against MBG improves renal function and attenuates renal fibrosis in a model of kidney disease (Haller et al., 2014). In addition, Cheng et al. (2019) found that targeting NKA-mediated signaling with pNaKtide (a peptide inhibiting NKA) markedly attenuated UUO-induced renal fibrosis. They further found that inhibition of Src activation and its downstream ERK1/2, p38 mitogen-activated protein kinase (MAPK) and AKT signaling pathways were the main mechanisms of the antifibrotic effects of pNaKtide. Telocinobufagin is a novel cardiotonic steroid with similar mechanistic effects in the kidney (Kennedy et al., 2018). Further study demonstrated that target-specific NKA was also effective. In a 5/6 nephrectomized rat model, using the NKA antagonist DRm217 alleviated glomerular atrophy and inhibited tubulointerstitial injury and fibrosis (Wang et al., 2019b). Therefore, targeting NKA inhibition is an important target for renal fibrosis (see Figure 3). CD40 receptor activation in the renal tubular epithelium is known to contribute to kidney injury and fibrosis (Haller et al., 2017). Recent studies have shown that knockdown of the NKA α1 heterodimer or loss of the functional NKA/Src cascade complex leads to a decrease in CD40, whereas rescue of the α1 heterodimer restores CD40 expression in renal epithelial cells (Xie et al., 2018), suggesting that NKA is a novel signaling mechanism for CD40 in the pathogenesis of renal injury and fibrosis.

Notably, in renal epithelial cells, RNAi-mediated specific knockdown of NKA-β1 induced loss of fibroblast phenotype, whereas ectopic expression of NKA-β1 reduced TGF-β1-mediated EMT, suggesting that NKA plays an important role in maintaining and shaping a well-differentiated phenotype of epithelial cells (Rajasekaran et al., 2010). Indeed, this may be related to the involvement of NKA-β1 in the assembly of tight junctions and the generation of epithelial cell polarity by including intracellular ion gradients, synergistic effects of E-cadherin, and regulation of MAPK, RhoA GTPase, and stress fibers (Rajasekaran and Rajasekaran, 2003).

### 4.3 NHE

Na^+^-H^+^ exchangers (NHEs) are membrane proteins widely present in mammals and are directly or indirectly involved in maintaining intracellular pH, cell volume regulation, cell proliferation migration and apoptosis (Orlowski and Grinstein, 1997). Nine well-characterized isoforms have been identified, of which NHE1-NHE5 are mainly located on the plasma membranes of different cell types and NHE6-NHE9 are restricted to intracellular organelles (Zhao et al., 2016). All NHE members appear to exist in the form of dimers, although the transfer function is in the form of monomers. NHE1 was the first to be identified, and in the kidney it is commonly expressed in the proximal tubule along the basolateral membrane of polarized epithelial cells (Coupaye-Gerard et al., 1996).

Apoptosis of renal tubular epithelial cells usually leads to tubular atrophy and renal fibrosis associated with CKD progression (Grgic et al., 2012; Schelling, 2016). It is well known that an important feature of apoptosis is a decrease in cell volume. Well-characterized ion channels and transporter proteins such as Cl^−^/HCO_3_^−^-exchanger and the Na^+^-K^+^-2Cl^−^ cotransporters (NKCC) are largely not expressed in the proximal tubule, making NHE1 particularly important as a regulatory volume increase (RVI)-mediated defense of renal tubular epithelial cells against apoptosis (Okada et al., 2001). Reduced expression of NHE1 in proximal tubules was noted in ureteral obstruction models (Manucha et al., 2007). A recent study found that fucoxanthin (extracted from brown seaweed) increased NHE1 expression, reduced tubular apoptosis and interstitial fibrosis, and improved renal function in CKD mice. This process involves the peroxisome activated receptor alpha (PPARα) pathway (Chen et al., 2018). The pro-survival effect of NHE1 has been demonstrated in various animal models of kidney disease (Wu et al., 2003; Khan et al., 2006; Manucha et al., 2007). In addition, Hydrogen ion extrusion-induced cytoplasmic alkalinization (Lagadic-Gossmann et al., 2004) and activation of the PI3K/Akt pathway induced by NHE1 (Wu et al., 2004) are equally important pro-survival mechanisms. As late NHE1 activity decreases, sustained apoptotic stimulation overcomes the pre-NHE1 effect, allowing the cells to move toward apoptosis and fibrosis.

In addition to facilitating Na^+^-H^+^ exchange and acting as a pro-survival factor, NHE1 is able to act as a molecular scaffolding platform to direct the formation of signaling complexes (Karydis et al., 2009). Indeed, NHEs are common targets of various inflammatory, oxidative stress stimuli and the increase in cell volume itself induces multiple changes in cell function and gene expression through the activation of osmotic signaling pathways (Lang et al., 1998). It was reported that NHE1 expression was stimulated after aldosterone treatment and that NHE1 was able to mediates aldosterone-associated fibronectin accumulation in rat mesangial cells through the ERK1/2 pathway, this direct effect could be ameliorated by shRNA-NHE1 (Zhang et al., 2010). Another study stresses the critical role of NHE-1 in an adriamycin (ADR)-induced glomerulosclerosis rat model, where NHE-1 mRNA expression was significantly enhanced, and high sodium diet accelerated interstitial fibrosis and further increased NHE1 expression, this effect could be prevented by amiloride (although not a specific NHE1 inhibitor) (Okuda et al., 1994). In addition, long-term lithium exposure is associated with chronic interstitial fibrosis, and the use of amiloride to partially reduce NHE1 activity can attenuate lithium-induced interstitial fibrosis, but the exact mechanism is not clear (Kalita-De Croft et al., 2018).

Interestingly, activated NHE1 is required for early cardiac hypertrophy and hepatic stellate cell proliferation in mice, yet its effect on renal fibroblasts has been poorly studied (Benedetti et al., 2001; Mraiche et al., 2011). Thus, we believe that NHE is a promising but understudied new direction for renal fibrosis research, since NHE1 can act as a predictor of fibrosis and targeting NHE1 does not inhibit basal exchange activity, which is the basis of ion homeostasis, but the mechanism needs to be better understood by more studies.

## 5 K^+^ channels

K^+^ channels are the most common and diverse superfamily of ion channels that are widely distributed in a variety of cell types and selectively allow the movement of K^+^ ions across the cell. K^+^ channels play an important role in the physiological and pathophysiological processes of cells, are involved in regulating cell proliferation, apoptosis, inflammation, immunity, and epithelial transport across a broad spectrum of the kidney (Welling, 2016). Importantly, K^+^ channels regulate resting membrane potential and therefore play a key role in cellular excitability. As an important contributor to setting membrane potential, K^+^ channels regulate cell cycle progression (membrane potential is not constant in the cell cycle, e.g., plasma membrane hyperpolarization occurs between G1 and S phases, while depolarization is necessary for cells to move from G2 to M) and help ensure the driving force of Ca^2+^ entry to influence cell cycle progression (Urrego et al., 2014). Dysregulation of the renal cell cycle is closely associated with renal fibrosis (Wu et al., 2021), especially renal tubular epithelial cells (TEC), and therefore we can speculate that K^+^ channels play an important role in renal fibrosis. Furthermore, as previously mentioned, the promotion of K^+^ efflux through K^+^ channels promotes the activation of NLRP3 inflammasome, which release inflammatory factors and thereby promote fibrogenesis (Solini et al., 2013; Di et al., 2018). There are generally four functional classes of K^+^ channels defined based on their structure, biophysical properties, and physiology: Ca^2+^-activated K^+^ channels (called KCa), voltage-gated K^+^ channels (called Kv), and others (inwardly rectifying K^+^ channels (K_ir_)and tandem pore domain K^+^ channels (K_2P_)channels) (Gonzalez et al., 2012).

### 5.1 Ca^2+^-activated K^+^ channels

As the name implies, KCa channels, a subgroup of K^+^ channels, are activated by intracellular micromolar Ca^2+^ and display different single-channel conductance to K^+^ ions, serving as an important link between cellular Ca^2+^ and electrical signaling (Rothberg, 2012). KCa channels are widely distributed in almost all cell types where they regulate a variety of cellular functions, including vascular tone, blood pressure, transmitter delivery, cell volume, membrane potential, and proliferation (Stocker, 2004). Based on their single channel conductance, the KCa channels were initially divided into three main types: large conductance Ca^2+^ -activated K^+^ channels (BK or KCa1.1), small (SK or KCa2.1–2.3), and intermediate conductance (IK or KCa3.1) (Wei et al., 2005).

Differences regarding the mechanisms of Ca^2+^ activation have revealed the division of KCa channels into two well-defined classes, as KCa1.1 is usually activated by the synergistic effect of voltage and cytoplasmic Ca^2+^ increase, while KCa2.1–2.3 and KCa3.1 channels, which share the same Ca^2+^/calmodulin (CaM)-mediated gating mechanism, are only gated by cytoplasmic Ca^2+^ increase (Fanger et al., 1999; Kaczmarek et al., 2017). To obtain the properties required for targeted activation, KCa channels are usually positioned close to physiological Ca^2+^ release sites, such as cell surface, endoplasmic reticulum storage release sites, and ultimately controlled by their switches. When a Ca^2+^ channel opens, the increased Ca^2+^ concentration activates all nearby KCa channels. The opening of KCa channels such as KCa3.1 regulates the rapid K^+^ efflux, leading to a high degree of hyperpolarization of the plasma membrane (a negative shift of the membrane potential towards the K^+^ equilibrium potential), which in turn increases the electrochemical driving force of Ca^2+^. Importantly this has been shown to occur during renal fibrosis, with increased KCa3.1 expression reportedly recorded from fibrotic kidneys of patients with diabetic nephropathy compared to normal (Huang et al., 2013), as well as KCa1.1 in fibrotic kidneys of mice (Wang et al., 2022). Here in this review we will discuss the intermediate conductance KCa3.1 channels and the large conductance BK channels that are most relevant to the particular topic.

#### 5.1.1 The KCa3.1 channel

**FIGURE 4:**
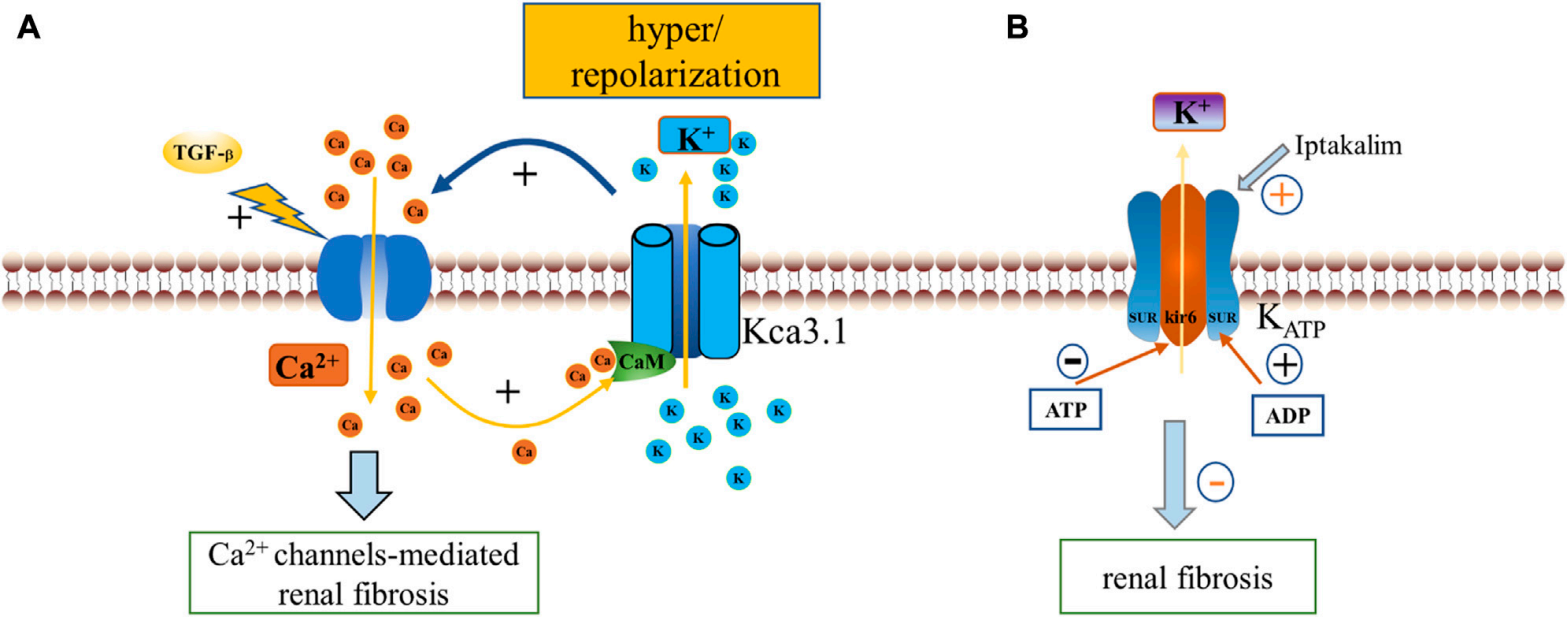
Diagram illustrating KCa3.1 channels activity regulating Ca^2+^ influx-dependent fibrosis pathway and regulation of K_ATP_ channels opening. **(A)**. KCa3.1 channels form a positive feedback loop with Ca^2+^ channels. TGF -β exposure promotes Ca^2+^ inward flow, and Ca^2+^ binding to the KCa3.1 channel CAM allosterically opens the channel, creating a membrane hyperpolarization or repolarization that further promotes Ca^2+^ inward flow, thereby triggering Ca^2+^ channels-mediated renal fibrosis. **(B)**. ATP binds to Kir6 subunits to inhibit the channel, while K_ATP_ opens in response to ADP dissociation at the SUR subunit. The iptakalim (a novel KATP channel opener) can open K_ATP_ channels to inhibit renal fibrosis. CAM, calmodulin; KCa3.1, Ca^2+^-activated K^+^ channel 3.1; K_ATP_, ATP-sensitive K^+^ channels.

The KCa3.1 channel is a tetrameric trans-membrane protein encoded by the gene KCNN4. Each subunit consists of six transmembrane structural domain with a pore between the fifth and sixth domain. The channel is insensitive to voltage due to the lack of a voltage-sensing structural domain, and is gated solely by internal Ca^2+^ ranging from 100 to 300 nM (Mcmanus, 1991; Ishii et al., 1997). Unlike KCa1.1 channels (binding directly to the channel), Ca^2+^ is normally bound to CaM, which is constitutively associated with the intracellular channel through the C-terminus, leading to a conformational change and subsequent opening of the KCa3.1 channel (Fanger et al., 1999). Functionally, KCa3.1 is involved in the regulation of membrane potential, Ca^2+^ signaling and cell volume in almost all cells. It was found that KCa3.1 channels play an important role in the RVD response in different cell types (Khanna et al., 1999; Wang et al., 2003), deletion of KCNN4 inhibits the ability of T lymphocytes and erythrocytes to regulate osmotic changes in mice (Begenisich et al., 2004), and as mentioned before, activation of KCa3.1 channels and the resulting K^+^ efflux would cause an exaggerated Ca^2+^ influx by hyperpolarization/repolarization of the plasma membrane and loss of electric potential against Ca^2+^ influx (see Figure 4A). KCa3.1-mediated Ca^2+^ influx is pathologically associated with various diseases including inflammation, atherosclerosis, autoimmune diseases, and cancer, and has been shown to play an important role in promoting mitogenic and proliferative features of tissues (Chou et al., 2008; Feske et al., 2015).

Recent studies have examined the role of KCa3.1 in mediating renal fibrosis. Grgic and colleagues showed that in mouse renal fibroblasts, mitogenic stimulation with the mitogen basic fibroblast growth factor (bFGF) upregulated KCa3.1 expression, and the KCa3.1-specific inhibitor TRAM-34 suppressed renal fibroblast proliferation in a dose-dependent manner through cell cycle arrest in the G0/G1 phase, which normally requires membrane hyperpolarization and increased Ca^2+^ inward flow (Grgic et al., 2009a). They also indicated that KCa3.1 was upregulated in fibrotic kidneys of mice induced by UUO, and again KCa3.1-deficient mice or mice treated with TRAM-34 markedly reduced the number of myofibroblasts and delayed renal fibrosis in mice after ureteral obstruction when compared to control mice (Grgic et al., 2009a). Huang et al. (2014a) further demonstrated that blockade of KCa3.1 attenuated renal fibrosis in a diabetic mouse model, and the downregulation of collagen synthesis, α-smooth muscle actin as well as the reduction in fibroblast activation supported this result. Importantly, in human renal interstitial fibroblasts, TRAM34 inhibited cell activation induced by TGF-β1 and also reduced the expression of fibrosis-related genes matrix metalloproteinase-2 (MMP2) and MMP9 (Huang et al., 2014a), suggesting a prominent role of targeting KCa3.1 in reversing renal fibroblast activation.

KCa3.1 plays an important role in TGF-β1 signaling and may be mediated through Smad3, P38, or ERK1/2 phosphorylation pathways (Huang et al., 2014b). Huang et al. (2014b) demonstrated that TGF-β1-induced current through KCa3.1 channels was inhibited by TRAM34 using patch clamp technique in human proximal tubule cells. Actually, TGF-β enhanced KCa3.1 activity, which in turn contributed to the activation of mitogen-activated protein kinase signaling and increased expression of monocyte chemoattractant protein-1 (MCP-1), which is critical to the pathogenesis of renal fibrosis (Huang et al., 2014b). Furthermore, both in human proximal tubule cells and in mouse kidney, high glucose-induced elevation of cytokine CCL20 expression and NF-κB binding activity were significantly associated with KCa3.1 (Huang et al., 2014c), suggesting that cytokine-induced renal injury may be mediated through modulation of K-channel activity. At the immune level, KCa3.1-mediated intracellular Ca^2+^ influx and membrane potential are required for T cell, macrophage and mast cell migration and inflammatory chemokine and cytokine production (Cruse et al., 2006; Toyama et al., 2008), and inhibition of KCa3.1 using TRAM34 reduces renal fibrosis in diabetic mice by inhibiting the generation of pro-inflammatory cytokines and macrophage infiltration (Huang et al., 2014c). KCa3.1 is also associated with TGF-β1-induced premature senescence and mesangial cell proliferation (Fu et al., 2014).

Taken together, these results suggest that KCa3.1 may represent a highly promising approach for the future treatment of renal tubulointerstitial fibrosis, and that inhibition of KCa3.1 may have more benefits than inhibition of myofibroblasts, possibly through direct effects on a variety of cells including fibroblasts, but also through indirect effects on inflammatory and immune cells.

#### 5.1.2 BK channels

BK channels, also called as KCa1.1, maxiK, slo1, are activated in response to elevated intracellular Ca^2+^ and membrane depolarization. Structurally BK channels are homotetramers in which the four α-subunits of the pore formation are encoded by the gene KCNMA1 (Lee and Cui, 2010). Each α subunit consists of seven transmembrane regions (S0-S6), with these different structural domains responsible for various functions. The S0 region at the N-terminus on the outer side of the membrane is usually associated with regulatory BKβ subunits, and the S1-S4 regions contain voltage sensors (with several positively charged residues) that make these channels sensitive to voltage. Four structural domains (S7- S10) carrying hydrophobic fragments extend from the intracellular C-terminus. the S7 and S8 domains form the regulator of conductance of K (RCK1) region, while the S9 and S10 regions allow Ca^2+^ binding in the so-called “Ca^2+^ bowl” (Cui et al., 2009; Ge et al., 2014). Activated K^+^ fluxes are variably modulated by intracellular voltage or ligand sensors through pore structures in response to various stimuli, thereby linking cellular signaling and membrane excitability.

A recent study indicates that BK channels play a key role in the prevention of renal fibrosis. Wang et al. (2022) reported that BK protein expression was reduced in renal tissue of UUO- or folate-induced fibrotic mice when compared to control mice, and that BK-deficient mice were more susceptible to renal fibrosis. Pharmacological activation of BK channels effectively prevented the development of renal fibrosis and protected renal function by inhibiting TGF-β/Smad signaling. Their further study of the mechanism revealed that BK channels could accelerate the degradation of TGF-β receptors through caveolae pathway, thereby achieving inhibition of the TGF-β signaling pathway (Wang et al., 2022). Inconsistent with these studies, BK channels appear to mediate glomerular pathological processes in high glucose cultured rat mesangial cells and induce the expression of collagen IV, fibronectin, TGF-β1 and Smad2/3 (Wu et al., 2020). This may be related to BK cell specificity as well as complex mechanisms. Studies on how BK channels alter epithelial cell responses to TGF-β *via* ion flux or downstream signaling mediators and which are the key cell types remain poorly understood.

### 5.2 Kv1.3

Kv1.3 is a member of the large and common voltage-dependent K^+^ channel (Kv) superfamily that is widely expressed in all organisms and is known to consist of several subfamilies (Kv1-4), of which Kv1.1-1.7 has been extensively studied. Kv1.3 was originally identified in T lymphocytes, and consists of four identical α subunits forming a homotetramer, each consisting of six transmembrane structural domains (S1-S6)and a P-loop. Among them, S4 segment is responsible for channel membrane voltage sensing, while S5 and S6 form a central pore structural domain, which has a highly sensitive selectivity for K^+^ ions (Perez-Verdaguer et al., 2016). Kv 1.3 is involved in a wide range of physiological and pathological processes, including Ca^2+^ signaling, cell volume regulation, cytokine secretion, energy homeostasis, cell proliferation and migration, and plays an important role in chronic inflammation, cancer progression, autoimmune diseases and other processes (Wulff et al., 2009; Serrano-Albarras et al., 2018; Wang et al., 2019c).

Kv1.3 is a key regulatory protein in the immune response and is highly expressed in activated effector T cells. Selective blockade of Kv1.3 channels modulates Ca^2+^ signaling influx patterns and inhibits T cell transcription and proliferation, thereby exerting immunosuppressive actions (Orban et al., 2013; Perez-Garcia et al., 2018). Kv1.3-mediated renal fibrosis is closely associated with inflammatory immune responses and fibrogenic cytokines. Previous studies have shown that Kv1.3 in T lymphocytes is involved in the pathogenesis of renal diseases, including acute glomerulonephritis, lupus nephritis and chronic kidney disease, and that its expression would contribute to disease progression (Grgic et al., 2009b; Hyodo et al., 2010; Kazama et al., 2012; Khodoun et al., 2020). In the advanced stages of congestive heart failure (CHF), upregulation of Kv1.3 channels activates the proliferation of regulatory T cells (Tregs), which promotes cardiac fibrosis by secreting the fibrotic cytokine TGF-β (Shao et al., 2018). Kazama et al. (2012), Kazama (2015) found diffuse interstitial fibrosis with leukocyte infiltration in the kidney of 5/6 nephrectomy rats, and Kv1.3 channel expression was upregulated in proliferating leukocytes in fibrotic kidneys. Importantly, inhibition of Kv1.3 channels using the selective blocker margatoxin significantly inhibited renal lymphocyte proliferation and ameliorated the progression of renal fibrosis. Their team further demonstrated the effectiveness of targeting lymphocyte Kv1.3 channels in the treatment of renal fibrosis using the UUO mouse model (Abe et al., 2019). Furthermore, stimulation with TGF-β significantly induced an increase in Kv1.3 density and outward K^+^ current amplitude, and the Kv1.3 channel inhibitor regulated the expression of the TGF-β in mouse microglia (Schilling et al., 2000), hypothesizing that Kv1.3 channel inhibitors have a similar mechanism to alleviate renal fibrosis by regulating the expression of the cytokine TGF-β.

In conclusion, it is necessary to conduct a more thorough study on the protective effect of Kv1.3 on renal fibrosis through the regulation of immune cell function to provide attractive ideas for the prevention and treatment of renal diseases.

### 5.3 ATP-sensitive K^+^ channels

The typical ATP-sensitive K^+^ (K_ATP_) channels are ubiquitous hetero-octameric complexs consisted of at least two proteins: the pore-forming subunit belonging to the Kir6.0 family (Kir6.1 or Kir6.2) and the sulfonyl binding regulatory subunit (SUR1 or SUR2) (Rodrigo and Standen, 2005; Tinker et al., 2018). Structurally, the tetramer composed by the kir6 family subunits forms a K^+^-selective pore-forming channel that is surrounded by four SUR protein subunits. Energy switch located at the cytoplasmic end of the channel lumen, where ATP binds to the Kir6 subunit to provide energy for channel closure, while SUR has an ADP binding site and ADP dissociation leads to a sustained “activation state” (Nichols et al., 2013). In other words, K_ATP_ channels open in response to a decrease in cellular ATP (see Figure 4B). However, cells ordinarily contain millimolar ATP, while channels open in response to micromolar concentrations, which makes channel opening rare. In this way, as endogenous metabolic sensors, K_ATP_ channels have been suggested to play a key role in matching membrane electrical excitability to the energy metabolic state, as well as in processes including maintenance of glucose homeostasis, energy, systemic blood pressure, and vascular tone regulation (Seino and Miki, 2003; Flagg et al., 2010). The evidence on the role of K_ATP_ in renal fibrosis is limited and remains of particular interest. Xue et al. (2005) reported that K_ATP_ channel subunit SUR2, Kir6.1 mRNA expression was upregulated in renal tissue of hypertensive rats. Iptakalim, a novel K_ATP_ channel opener, regulated Kir6.1 overexpression (no effect on SUR2) and inhibited the accumulation of extracellular matrix components [fibronectin, collagen IV, MMP-9 and tissue inhibitor of MMP 1 (TIMP-1)] in the kidney of hypertensive rats, protecting renal function and preventing fibrosis progression. They also found that this effect was accompanied by a decrease in the expression of TGF-β1, endothelin 1 in the kidney during hypertensio (Xue et al., 2005). Despite the limited data, we believe that K_ATP_ is a promising new direction for research in renal fibrosis and look forward to new research findings in this area.

## 6 Conclusion

Undoubtedly, years of research established that studies regarding physiology, pathology and mechanism of ion channels in renal fibrosis have gradually become a hotspot in our description, and the feasibility of ion channels in the treatment and intervention of diseases has been confirmed by an increasing number of trials. In addition to their function as channels, the interactions between ion channels and proteins indicate their involvement in different processes such as proliferation, apoptosis, atrophy, cell cycle, cell acidity, cytoskeletal structure, immune and cell volume regulation, all of which are related to renal fibrosis. Although efforts to characterize how ion channels perform these important functions have somewhat provided us with mechanistic insight, there are still numerous unanswered questions and even controversy. It is important to emphasize that chloride channels are a promising and novel direction in renal fibrosis research, but the potential relationship between them and intracellular acidification, how they connect as channel regulators and integrate cellular signaling and function still needs to be catalogued. The availability of new pharmacology based on the genetic properties, structural differences and gating mechanisms of different chloride channels may allow for more successful therapies in the future. Although STIM and Orai have emerged as core elements of highly evolutionarily conserved Ca^2+^ channels, there are still large gaps in signaling pathways, isoform differences as well as cell and tissue specificity. There is an urgent need to develop specific drugs targeting the exciting P2X7/TRPC channel, however, research on the differential regulatory mechanisms of different subtypes and drug development are still worth exploring. The role of Na channels and transporters in the renal fibrosis pathway should not be ignored. We are looking forward to the pharmacological and genetic targeting studies of ENaC protein on renal fibrosis. An equally exciting pathways in renal fibrosis is potassium handling; in the future, hopefully more K^+^ channel agonists or inhibitors will be used in this area. It must be emphasized that the crosstalk between various ion channels is complex and the coordinated functioning of the entire channel network determines ion homeostasis in the kidney, which is reflected in the complex pathological features of most renal diseases. Understanding the rules of channel interactions remains an important challenge for successful intervention in various forms of renal fibrosis. In addition, the abundance of scientific research tools is essential for the accurate measurement of channel regulation within some cells. At last, since ion channels are widely expressed in most cells and tissues, drugs designed for channel-related diseases should focus more on tissue and subtype specificity to avoid safety issues, and precise intracellular delivery of novel modulators based on nanoplatforms might be a promising option. Overall, continually refining our understanding of the ion channel transport mechanisms that underlie renal fibrosis will undoubtedly expand therapeutic possibilities, and more research is needed to decipher the complex chain of events in renal fibrosis.
